# Regio- and Stereoselective Allylindation of Alkynes Using InBr_3_ and Allylic Silanes: Synthesis, Characterization, and Application of 1,4-Dienylindiums toward Skipped Dienes

**DOI:** 10.3390/molecules23081884

**Published:** 2018-07-27

**Authors:** Yoshihiro Nishimoto, Junyi Yi, Tatsuaki Takata, Akio Baba, Makoto Yasuda

**Affiliations:** 1 Frontier Research Base for Global Young Researchers Center for Open Innovation Research and Education (COiRE), Graduate School of Engineering, Osaka University; Osaka 565-0871, Japan; 2 Department of Applied Chemistry, Graduate School of Engineering, Osaka University; Osaka 565-0871, Japan; j_yi@chem.eng.osaka-u.ac.jp (J.Y.); tatsu_takata@chem.eng.osaka-u.ac.jp (T.T.); baba@chem.eng.osaka-u.ac.jp (A.B.)

**Keywords:** indium, allylmetalation, alkyne, allylic silane

## Abstract

Regioselective *anti*-allylindation of alkynes was achieved using InBr_3_ and allylic silanes. Various types of alkynes and allylic silanes were applicable to the present allylindation. This sequential process used the generated 1,4-dienylindiums to establish novel synthetic methods for skipped dienes. The 1,4-dienylindiums were characterized by spectral analysis and treated with I_2_ to stereoselectively give 1-iodo-1,4-dienes. The Pd-catalyzed cross coupling of 1,4-dienylindium with iodobenzene successfully proceeded in a one-pot manner to afford the corresponding 1-aryl-1,4-diene.

## 1. Introduction

**Scheme 1:**
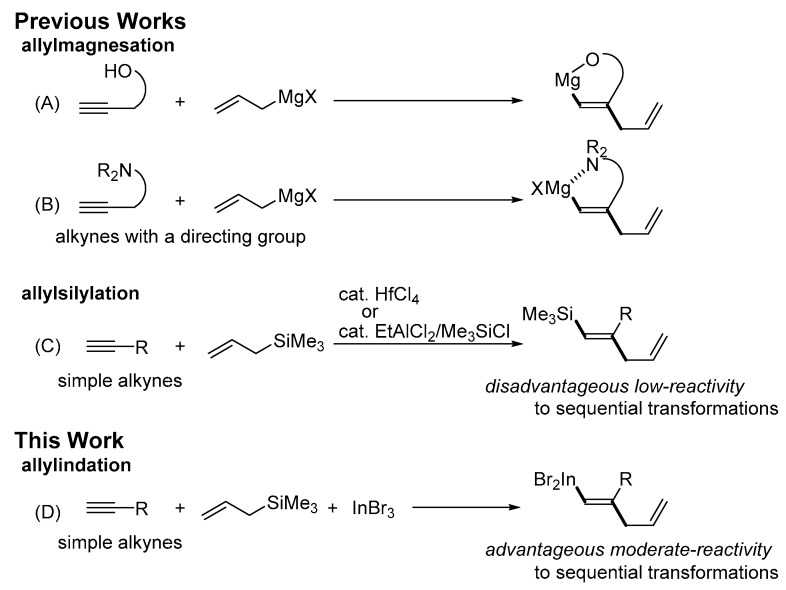
*Anti*-allylmetalation of alkynes.

Carbometalation is an important synthetic method in organic synthesis because organometallic compounds are produced with an expansion of the carbon framework [1,2,3,4,5,6,7]. In particular, the allylmetalation of alkynes provides metalated skipped dienes (1,4-diene), which are effectively transformed to functionalized skipped dienes via sequential reactions [8,9,10,11,12,13,14,15,16,17,18]. Skipped diene units are present in many biologically important natural products, and are also versatile synthetic building blocks in organic synthesis [19,20,21,22]. Therefore, various types of allylmetalation of alkynes have been developed. However, most reported reactions involve a *syn*-addition to alkynes, and few reports have focused on *anti*-allylmetalation (Scheme 1). Allylmagnesations via direct *anti*-addition of allylic Grignard reagent were also reported (Scheme 1A,B), in which a directing group such as hydroxy and amino groups nearby the alkyne moiety are required [23,24,25,26,27,28,29]. Yamamoto reported an allylsilylation of simple alkynes with allylic silanes catalyzed by either HfCl_4_ or EtAlCl_2_-Me_3_SiCl (Scheme 1C) [16,30,31,32]. However, the produced 1,4-dienyl trialkylsilanes cannot be applied to sequential transformations such as Hiyama coupling without activation by a strong base because of their low reactivity. In this context, we achieved regioselective *anti*-allylindation of simple alkynes using InBr_3_ and allylic silanes (Scheme 1D). To the best of our knowledge, *anti*-allylindation of alkynes has never been established, while several *syn*-allylindations using allylic indiums have [13,33,34,35,36,37,38,39]. The 1,4-dienylindium compounds can be excellent precursors for functionalized skipped dienes due to their moderate reactivity and high compatibility with many functional groups. In fact, the 1,4-dienylindiums synthesized by the present allylindation can be easily transformed to functionalized skipped dienes by iodination or Pd-catalyzed cross coupling without the addition of bases in contrast to 1,4-dienylsilanes produced via allylsilylation.

## 2. Results

Recently, we reported regioselective *anti*-carbometalations of alkynes using organosilicon nucleophiles and metal halides such as InBr_3_ [40], GaBr_3_ [41], BiBr_3_ [42], ZnBr_2_ [43], and AlBr_3_ [44]. In our established carbometalations, a metal halide directly activates an alkyne, and then an organosilicon nucleophile adds to the alkyne from an opposite site of the metal halide. Therefore, we applied a combination of indium trihalides and allylic silanes to establish *anti*-allylindation of alkynes. First, various indium salts were investigated for the reaction using alkyne **1a** and methallyl trimethylsilane **2a** (Table 1). InBr_3_, **1a**, and **2a** were mixed in CH_2_Cl_2_, and then the reaction mixture was stirred at room temperature for 24 h. After an I_2_ solution in THF was added at −78 °C, alkenyl iodide **4aa** was obtained as a single isomer in 89% yield (Entry 1). An iodine group was introduced exclusively cis to the allylic group. The production of **4aa** by quenching with I_2_ suggested that *anti*-allylindation regioselectively proceeded to give the corresponding 1,4-dienylindium **3aa**. The use of InCl_3_ instead of InBr_3_ afforded **4aa** in a 42% yield (Entry 2). On the other hand, examinations using InF_3_, InI_3_, and In(OTf)_3_ resulted in no reaction (Entries 3–5). The thermodynamic stability of a generated side product Me_3_SiX might influence the driving force of the reaction. An investigation of the solvent effect was carried out. The reaction performed in non-polar solvents such as toluene resulted in no product because InBr_3_ did not dissolve the solvent (Entry 6). Polar solvents such as Et_2_O, CH_3_CN, and THF were not suitable to the present allylindation because of the deactivation of InBr_3_ by the solvent coordination (Entries 7–9).

The scope of the alkynes **1** is shown in Table 2. Sterically hindered aliphatic alkynes **1b** and **1c** (R = primary alkyl group) that were slightly larger than **1a** resulted in lower yields of the corresponding alkenyl iodides **4ba** and **4ca**, respectively (Entries 1 and 2). Cyclohexylacetylene **1d** (R = secondary alkyl group) gave a moderate yield (Entry 3), and the allylindation of *tert*-butylacetylene **1e** did not proceed due to large steric hindrance (Entry 4). These results showed that the steric hindrance on an alkyne disturbs the allylindation. This allylindation system tolerated functionalities such as Ph and alkyl chloride moieties (Entries 5 and 6). Aromatic alkyne **1h** was also applicable to the present allylindation. In this case, the addition of Me_2_Si(OMe)_2_ effectively increased the yield of the desired alkenyl iodide **4ha** (Entries 7 and 8), probably because the MeO group of Me_2_Si(OMe)_2_ coordinated to an indium atom of the produced 1,4-dienylindium **3** to stabilize **3,** and to avoid protonation of **3** by alkyne **1h**.

Next, we evaluated the scope of allylic silanes **2** in the allylindation of alkyne **1h** in the presence of Me_2_Si(OMe)_2_ (Table 3). Allylindation using the simplest allylic silane **2b** effectively proceeded to give the desired product **4hb** in 48% yield (Entry 1). Allylic silane **2c** bearing a Ph group at the 2-position also afforded a high yield (Entry 2). Allylindations using prenylsilane **2d** and cinnamylsilane **2e**, which have a substituent at the 3-position, effectively occurred to give the corresponding iodinated skipped dienes **4hd** and **4he** in 72% and 39% yields, respectively (Entries 3 and 4).

**Figure 1:**
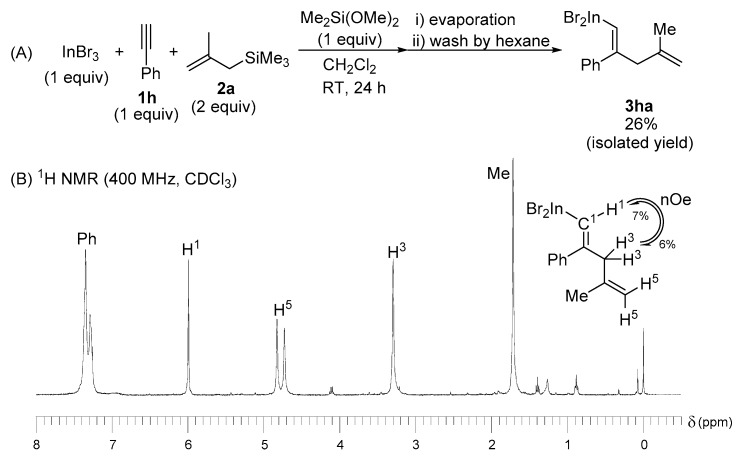
Isolation and characterization of 1,4-dienylindium synthesized by allylindation. (**A**) Isolation of 1,4-dienylindium **3ha**. (**B**) 1H-NMR spectrum of **3ha**.

The 1,4-dienylindium **3** synthesized by the present allylindation were isolated and characterized (Figure 1). After the allylindation of alkyne **1h** using InBr_3_ and methallylsilane **2a**, the volatiles were evaporated and the residual oil was washed with hexane to obtain the desired 1,4-dienylindium **3ha** as a white solid (Figure 1A). The 1,4-dienylindium **3ha** was characterized by NMR spectroscopy. The resonance of a vinylic proton (H^1^) at the α-position of the InBr_2_ group appeared at δ 5.99 ppm (Figure 1B). The ^13^C-NMR spectrum of **3ha** showed a slightly broad signal for C^1^ at δ 134.1 ppm. These chemical shift values are similar to those of previously reported alkenylindium generated by the carboindation of alkyne **1h** with InBr_3_ and a silyl ketene acetal [41]. A nuclear Overhauser effect between H^1^ and H^3^ was observed, which showed that *anti*-allylindation proceeded stereoselectively to give 1,4-dienylindium with a *trans*-configuration between the InBr_2_ and allylic groups.

**Scheme 2:**
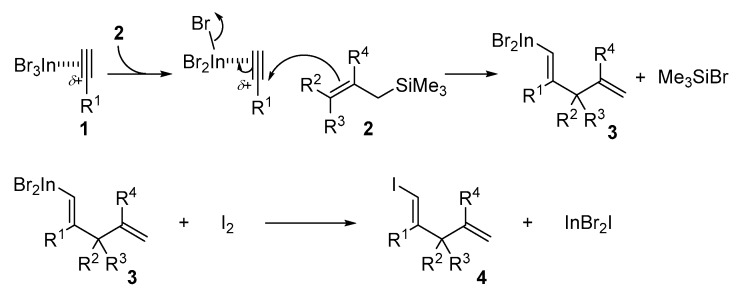
Plausible reaction mechanism.

A plausible reaction mechanism is illustrated in Scheme 2. A carbon-carbon triple bond of alkyne **1** coordinates to InBr_3_, and then the positive charge on the internal carbon atom of alkyne **1** is increased. Allylic silane **2** adds to the internal carbon atom from the opposite side of InBr_3_ to give 1,4-dienylindium **3**. The iodination of 1,4-dienylindium **3** with I_2_ proceeds with retention of the double bond configuration of **3** to yield alkenyl iodide **4** as a single isomer.

**Scheme 3:**
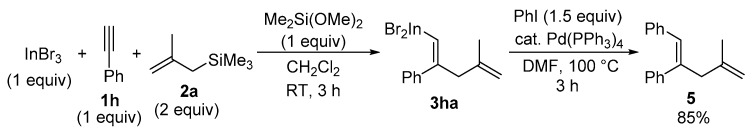
Pd-catalyzed cross-coupling of alkenylindium with iodobenzene.

Finally, we applied the synthesized 1,4-dienylindium to Pd-catalyzed cross coupling [40,45,46]. After 1,4-dienylindium **3ha** was produced via the allylindation of alkyne **1h** with allyl silane **2a** and InBr_3_, iodobenzene, a catalytic amount of Pd(PPh_3_)_4_, and DMF were added to the reaction mixture in a one-pot manner. Then, the Pd-catalyzed coupling reaction of **3ha** with iodobenzene smoothly proceeded at 100 °C to give the desired skipped diene **5** as a single isomer. It should be noted that the coupling product **5** was stereoselectively obtained with retention of the double bond configuration of the alkenylindium (Scheme 3).

## 3. Materials and Methods

### 3.1. Analysis

NMR spectra were recorded on a JEOL JNM-400 (400 MHz for ^1^H-NMR and 100 MHz for ^13^C-NMR) spectrometer (JEOL Ltd., Tokyo, Japan). Chemical shifts were reported in ppm on the δ scale relative to tetramethylsilane (δ = 0 for ^1^H-NMR) with the residual CHCl_3_ (δ = 77.0 for ^13^C-NMR) used as an internal reference. ^1^H and ^13^C-NMR signals of all new compounds were assigned by using HMQC, HMBC, COSY, and ^13^C off-resonance techniques. Infrared (IR) spectra were recorded on a JASCO FT/IR-6200 Fourier transform infrared spectrophotometer (JASCO Co., Tokyo, Japan). Silica gel column chromatography was performed using an automated flash chromatography system from the Yamazen Co. (W-Prep 2XY) (Yamazen Co., Osaka, Japan). Gel permeation chromatography (GPC) was performed using a NEXT recycling preparative HPLC from the Japan Analytical Industry Co. (Tokyo, Japan) (solvent: CHCl_3_; column: JAIGEL-1HH and JAIGEL-2HH). Reactions were carried out in dry solvents under a nitrogen atmosphere, unless otherwise stated. All allylic silanes were prepared by reported methods. Other reagents were purchased from Sigma-Aldrich Co. (St. Louis, MO, USA), the Tokyo Chemical Industry Co., Ltd. (TCI) (Tokyo, Japan) or Wako Pure Chemical Industries, Ltd. (Osaka, Japan), and used after purification by distillation or used without purification for solid substrates.

### 3.2. Typical Procedure

Alkyne **1** (1 mmol) was added to a solution of InBr_3_ (1 mmol) and allylic silane **2** (2 mmol) in dichloromethane (1 mL). The mixture was stirred at room temperature for 24 h, and then 0.75 M I_2_ in THF solution (2 mL) was added at −78 °C. The resultant mixture was stirred at −78 °C for 30 min. The mixture was quenched by saturated Na_2_S_2_O_3_ aq (10 mL), and then extracted with dichloromethane (3 × 10 mL). The collected organic layers were dried over MgSO_4_, and concentrated under reduced pressure. The yield was determined by ^1^H-NMR using 1,1,2,2-tetrachloroethane as an internal standard. The crude product was purified by flash chromatography (spherical silica gel 60 μm, 30 g, diameter 2.7 cm) and GPC to give the product.

*(E)-4-(Iodomethylene)-2-methyldodec-1-ene* (**4aa**)


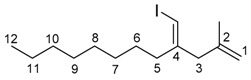


The alkyne 1-decyne (0.980 mmol, 0.1354 g) was added to a solution of InBr_3_ (0.996 mmol, 0.3530 g) and methallyl trimethylsilane (2.07 mmol, 0.2654 g) in dichloromethane (1 mL). The mixture was stirred at room temperature for 24 h. The reaction mixture was cooled to −78 °C, and 0.75 M I_2_ in THF solution (2 mL) was added. The resultant mixture was stirred at −78 °C for 30 min. The mixture was quenched by saturated Na_2_S_2_O_3_ aq (10 mL). The mixture was extracted with dichloromethane (3 × 10 mL). The collected organic layer was dried over MgSO_4_. The solvent was evaporated, and the residue was purified by column chromatography (hexane, column length 10 cm, diameter 26 mm silica gel) and GPC (CHCl_3_) to give the product (0.279 g, 89%).

IR: (neat) 1650, 1457 cm^−1^; ^1^H-NMR: (400 MHz, CDCl_3_) 5.92 (s, 1H, 4-CHI), 4.83 (s, 1H, 1-H), 4.75 (s, 1H, 1-H), 2.87 (s, 2H, 3-H_2_), 2.16 (t, *J* = 7.8 Hz, 2H, 5-H), 1.65 (s, 3H, 2-Me), 1.43–1.23 (m, 14H), 0.88 (t, *J* = 6.8 Hz, 3H); ^13^C-NMR: (100 MHz, CDCl_3_) 149.4 (s, C-4), 142.5 (s, C-2), 113.0 (t, C-1), 76.2 (d, 4-CHI), 45.8 (t, C-3), 36.4 (t, C-5), 31.9 (t), 29.43 (t), 29.38 (t), 29.22 (t), 27.0 (t), 22.7 (t), 21.8 (q, 2-Me), 14.1 (q, C-12); HRMS: (EI, 70 eV) Calculated (C_14_H_25_I) 320.1001 (M^+^), Found: 320.1000.

*(E)-4-(Iodomethylene)-2,7-dimethyloct-1-ene* (**4ba**)


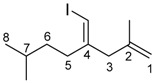


The alkyne 5-methylhex-1-yne (1.02 mmol, 0.0985 g) was added to a solution of InBr_3_ (0.983 mmol, 0.3485 g) and methallyl trimethylsilane (1.94 mmol, 0.2487 g) in dichloromethane (1 mL). The mixture was stirred at room temperature for 24 h. The reaction mixture was cooled to −78 °C, and 0.75 M I_2_ in THF solution (2 mL) was added. The resultant mixture was stirred at −78 °C for 30 min. The mixture was quenched by saturated Na_2_S_2_O_3_ aq (10 mL). The mixture was extracted with dichloromethane (3 × 10 mL). The collected organic layer was dried over MgSO_4_. The solvent was evaporated and the residue was purified by column chromatography (hexane, column length 10 cm, diameter 26 mm silica gel) and GPC (CHCl_3_) to give the product (0.0930 g, 33%).

IR: (neat) 1650, 1467, 1455 cm^−1^; ^1^H-NMR: (400 MHz, CDCl_3_) 5.90 (s, 1H, 4-CHI), 4.83 (s, 1H, 1-H), 4.75 (s, 1H, 1-H), 2.88 (s, 2H, 3-H_2_), 2.18–2.16 (m, 2H, 5-H_2_), 1.65 (s, 3H, 2-Me), 1.62–1.52 (m, 1H, 7-H), 1.30–1.24 (m, 2H, 6-H_2_), 0.93 (d, *J* = 6.3 Hz, 6H, 8-H_3_ and 7-Me); ^13^C-NMR: (100 MHz, CDCl_3_) 149.5 (s, C-4), 142.4 (s, C-2), 113.1 (t, C-1), 75.9 (d, 4-CHI), 45.8 (t, C-3), 36.0 (t, C-6), 34.5 (t, C-5), 28.2 (d, C-7), 22.5 (q, C-8 and 7-Me), 21.8 (q, 2-Me); HRMS: (EI, 70 eV) Calculated (C_11_H_19_I) 278.0531 (M^+^), Found: 278.0529.

*(E)-4-(Iodomethylene)-2,6-dimethylhept-1-ene* (**4ca**) 


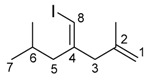


The alkyne 4-methylpent-1-yne (1.06 mmol, 0.0872 g) was added to a solution of InBr_3_ (1.02 mmol, 0.3606 g) and methallyl trimethylsilane (2.03 mmol, 0.2620 g) in dichloromethane (1 mL). The mixture was stirred at room temperature for 24 h. The reaction mixture was cooled to −78 °C, and 0.75 M I_2_ in THF solution (2 mL) was added. The resultant mixture was stirred at −78 °C for 30 min. The mixture was quenched by saturated Na_2_S_2_O_3_ aq (10 mL). The mixture was extracted with dichloromethane (3 × 10 mL). The collected organic layer was dried over MgSO_4_. The solvent was evaporated and the residue was purified by column chromatography (hexane, column length 10 cm, diameter 26 mm silica gel) and GPC (CHCl_3_) to give the product (0.0560 g, 20%).

IR: (neat) 1650, 1463 cm^−1^; ^1^H-NMR: (400 MHz, CDCl_3_) 6.01 (s, 1H, 8-H), 4.84 (s, 1H, 1-H), 4.74 (s, 1H, 1-H), 2.87 (s, 2H, 3-H_2_), 2.09 (d, *J* = 8.0 Hz, 2H, 5-H_2_), 1.90 (septet, *J* = 8.0 Hz, 1H, 6-H), 1.65 (s, 3H, 2-Me), 0.93 (d, *J* = 0.8 Hz, 6H, 7-H_3_ and 6-Me); ^13^C-NMR: (100 MHz, CDCl_3_) 148.4 (s, C-4), 142.4 (s, C-2), 113.2 (t, C-1), 77.5 (d, C-8), 46.1 (t, C-3), 44.7 (t, C-5), 26.8 (d, C-6), 22.4 (q, C-7 and 6-Me), 21.8 (q, 2-Me); HRMS: (EI, 70 eV) Calculated (C_10_H_17_I) 264.0375 (M^+^), Found: 264.0370.

*(Z)-(1-Iodo-4-methylpenta-1,4-dien-2-yl)cyclohexane* (**4da**)


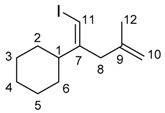


Ethynylcyclohexane (1.01 mmol, 0.1094 g) was added to a solution of InBr_3_ (0.968 mmol, 0.3432 g) and methallyl trimethylsilane (1.98 mmol, 0.2540 g) in dichloromethane (1 mL). The mixture was stirred at room temperature for 24 h. The reaction mixture was cooled to −78 °C, and 0.75 M I_2_ in THF solution (2 mL) was added. The resultant mixture was stirred at −78 °C for 30 min. The mixture was quenched by saturated Na_2_S_2_O_3_ aq (10 mL). The mixture was extracted with dichloromethane (3 × 10 mL). The collected organic layer was dried over MgSO_4_. The solvent was evaporated and the residue was purified by column chromatography (hexane, column length 10 cm, diameter 26 mm silica gel) and GPC (CHCl_3_) to give the product (0.0607 g, 21%).

IR: (neat) 1650, 1448 cm^−1^; ^1^H-NMR: (400 MHz, CDCl_3_) 5.83 (s, 1H, 11-H), 4.87 (s, 1H, 10-H), 4.77 (s, 1H, 10-H), 2.81 (s, 2H, 8-H_2_), 2.63–2.56 (m, 1H, 1-H), 1.79–1.55 (m, 8H), 1.4–1.23 (m, 4H), 1.20–1.09 (m, 1H); ^13^C-NMR: (100 MHz, CDCl_3_) 151.8 (s, C-7), 142.8 (s, C-9), 113.7 (t, C-10), 76.0 (d, C-11), 47.3 (d, C-1), 42.1 (t, C-8), 29.9 (t), 26.3 (t), 26.0 (t), 22.0 (t, C-12); HRMS: (EI, 70 eV) Calculated (C_12_H_19_I) 290.0531 (M^+^), Found: 290.0530.

*(E)-(3-(Iodomethylene)-5-methylhex-5-en-1-yl)benzene* (**4fa**) 


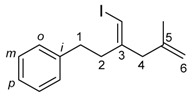


Pent-4-yn-1-ylbenzene (1.01 mmol, 0.1314 g) was added to a solution of InBr_3_ (0.979 mmol, 0.3471 g) and methallyl trimethylsilane (2.00 mmol, 0.2560 g) in dichloromethane (1 mL). The mixture was stirred at room temperature for 24 h. The reaction mixture was cooled to −78 °C, and 0.75 M I_2_ in THF solution (2 mL) was added. The resultant mixture was stirred at −78 °C for 30 min. The mixture was quenched by saturated Na_2_S_2_O_3_ aq (10 mL). The mixture was extracted with dichloromethane (3 × 10 mL). The collected organic layer was dried over MgSO_4_. The solvent was evaporated and the residue was purified by column chromatography (hexane, column length 10 cm, diameter 26 mm silica gel) and GPC (CHCl_3_) to give the product (0.1357 g, 43%).

IR: (neat) 1649, 1604, 1494, 1454 cm^−1^; ^1^H-NMR: (400 MHz, CDCl_3_) 7.31–7.17 (m, 5H, Ph), 6.00 (s, 1H, 3-CHI), 4.85 (s, 1H, 6-H), 4.76 (s, 1H, 6-H), 2.86 (s, 2H, 4-H_2_), 2.72–2.68 (m, 2H, 1-H_2_), 2.48–2.44 (m, 2H, 2-H_2_), 1.64 (s, 3H, 5-Me); ^13^C-NMR: (100 MHz, CDCl_3_) 148.4 (s, C-3), 142.2 (s, C-5), 141.4 (s, *i*), 128.39 (d), 128.35 (d), 126.0 (d, *p*), 113.3 (t, C-6), 77.2 (d, 3-CHI), 46.3 (t, C-4), 38.6 (t, C-2), 33.3 (t, C-1), 21.8 (q, 5-Me); HRMS: (EI, 70 eV) Calculated (C_14_H_17_I) 312.0375 (M^+^), Found: 312.0377.

*(E)-7-Chloro-4-(iodomethylene)-2-methylhept-1-ene* (**4ga**)


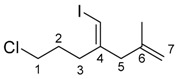


The alkyne 5-chloropent-1-yne (1.01 mmol, 0.1031 g) was added to a solution of InBr_3_ (0.983 mmol, 0.3486 g) and methallyl trimethylsilane (1.99 mmol, 0.2557 g) in dichloromethane (1 mL). The mixture was stirred at room temperature for 24 h. The reaction mixture was cooled to −78 °C, and 0.75 M I_2_ in THF solution (2 mL) was added. The resultant mixture was stirred at −78 °C for 30 min. The mixture was quenched by saturated Na_2_S_2_O_3_ aq (10 mL). The mixture was extracted with dichloromethane (3 × 10 mL). The collected organic layer was dried over MgSO_4_. The solvent was evaporated and the residue was purified by column chromatography (hexane, column length 10 cm, diameter 26 mm silica gel) to give the product (0.1676 g, 59%).

IR: (neat) 1649, 1443 cm^−1^; ^1^H-NMR: (400 MHz, CDCl_3_) 6.01 (s, 1H, 4-CHI), 4.84 (s, 1H, 7-H), 4.76 (s, 1H, 7-H), 3.54 (t, *J* = 7.3 Hz, 2H, 1-H_2_), 2.88 (s, 2H, 5-H_2_), 2.31 (t, *J* = 7.3 Hz, 2H, 3-H_2_), 1.88 (quintet, *J* = 7.3 Hz, 2H, 2-H_2_), 1.64 (s, 3H, 6-Me); ^13^C-NMR: (100 MHz, CDCl_3_) 147.6 (s, C-4), 141.9 (s, C-6), 113.4 (t, C-7), 77.6 (d, 4-CHI), 46.0 (t, C-5), 44.5 (t, C-1), 33.9 (t, C-3), 30.0 (t, C-2), 21.7 (q, 6-Me); HRMS: (EI, 70 eV) Calculated (C_9_H_14_ClI) 283.9829 (M^+^), Found: 283.9823.

*(Z)-(1-Iodo-4-methylpenta-1,4-dien-2-yl)benzene* (**4ha**)


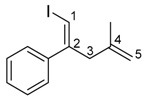


Phenylacetylene (1.08 mmol, 0.110 g) was added to a solution of InBr_3_ (1.00 mmol, 0.3541 g), methallyl trimethylsilane (1.99 mmol, 0.2552 g), and Me_2_Si(OMe)_2_ (1.02 mmol, 0.1230 g) in dichloromethane (1 mL). The mixture was stirred at room temperature for 24 h. The reaction mixture was cooled to −78 °C, and 0.75 M I_2_ in THF solution (2 mL) was added. The resultant mixture was stirred at −78 °C for 30 min. The mixture was quenched by saturated Na_2_S_2_O_3_ aq (10 mL). The mixture was extracted with dichloromethane (3 × 10 mL). The collected organic layer was dried over MgSO_4_. The solvent was evaporated and the residue was purified by column chromatography (hexane, column length 10 cm, diameter 26 mm silica gel) and GPC (CHCl_3_) to give the product (0.169 g, 55%).

IR: (neat) 1650, 1490, 1442 cm^−1^; ^1^H-NMR: (400 MHz, CDCl_3_) 7.38–7.30 (m, 3H, Ar), 7.21 (d, *J* = 6.8 Hz, 2H, Ar), 6.35 (s, 1H, 1-H), 4.78 (s, 1H, 5-H), 4.66 (s, 1H, 5-H), 3.20 (s, 2H, 3-H), 1.70 (s, 3H, 4-Me); ^13^C-NMR: (100 MHz, CDCl_3_) 150.1 (s), 141.9 (s), 141.5 (s), 128.1 (d), 127.9 (d), 127.6 (d), 113.7 (t, C-5), 77.6 (d, C-1), 48.6 (t, C-3), 21.9 (q, 4-Me); Calculated (C_12_H_13_I) 284.0062 (M^+^), Found: 284.0062.

*(Z)-(1-Iodopenta-1,4-dien-2-yl)benzene* (**4hb**)


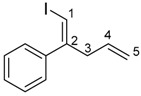


Phenylacetylene (1.00 mmol, 0.102 g) was added to a solution of InBr_3_ (1.11 mmol, 0.3921 g), allyl trimethylsilane (1.96 mmol, 0.2236 g), and Me_2_Si(OMe)_2_ (1.00 mmol, 0.1202 g) in dichloromethane (1 mL). The mixture was stirred at room temperature for 24 h. The reaction mixture was cooled to −78 °C, and 0.75 M I_2_ in THF solution (2 mL) was added. The resultant mixture was stirred at −78 °C for 30 min. The mixture was quenched by saturated Na_2_S_2_O_3_ aq (10 mL). The mixture was extracted with dichloromethane (3 × 10 mL). The collected organic layer was dried over MgSO_4_. The solvent was evaporated and the residue was purified by column chromatography (hexane, column length 10 cm, diameter 26 mm silica gel) and GPC (CHCl_3_) to give the product (0.102 g, 38%).

IR: (neat) 1638 cm^−1^; ^1^H-NMR: (400 MHz, CDCl_3_) 7.42–7.7.31 (m, 3H, Ar), 7.24–7.22 (m, 2H, Ar), 6.35 (t, *J* = 1.5 Hz, 1H, 1-H), 5.78 (m, 1H, 4-H), 5.11–5.06 (m, 2H, 5-H), 3.25 (dq, *J* = 6.8, 1.5 Hz, 2H, 3-H_2_); ^13^C-NMR: (100 MHz, CDCl_3_) 150.8 (s, C-2), 142.1 (s), 134.3 (d, C-4), 128.2 (d), 127.8 (d), 127.6 (d), 117.5 (t, C-5), 77.1 (d, C-1), 44.3 (t, C-3); HRMS: (EI, 70 eV) Calculated (C_11_H_11_I) 269.9905 (M^+^), Found: 269.9903.

*(Z)-1-Iodo-2,4-diphenylpenta-1,4-diene* (**4hc**)


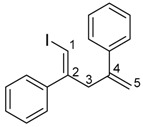


Phenylacetylene (1.03 mmol, 0.1053 g) was added to a solution of InBr_3_ (0.98 mmol, 0.3497 g), 2-phenylallyl trimethylsilane (2.00 mmol, 0.3813 g), and Me_2_Si(OMe)_2_ (1.00 mmol, 0.1207 g) in dichloromethane (1 mL). The mixture was stirred at room temperature for 24 h. The reaction mixture was cooled to −78 °C, and 0.75 M I_2_ in THF solution (2 mL) was added. The resultant mixture was stirred at −78 °C for 30 min. The mixture was quenched by saturated Na_2_S_2_O_3_ aq (10 mL). The mixture was extracted with dichloromethane (3 × 10 mL). The collected organic layer was dried over MgSO_4_. The solvent was evaporated and the residue was purified by column chromatography (hexane, column length 10 cm, diameter 26 mm silica gel) and GPC (CHCl_3_) to give the product (0.153 g, 43%).

IR: (neat) 1626, 1492, 1442 cm^−1^; ^1^H-NMR: (400 MHz, CDCl_3_) 7.36–7.23 (m, 8H, Ar), 7.17–7.15 (m, 2H, Ar), 6.33 (s, 1H, 1-H), 5.41 (s, 1H, 5-H), 5.07 (s, 1H, 5-H), 3.65 (s, 2H, 3-H_2_); ^13^C-NMR: (100 MHz, CDCl_3_) 149.9 (s), 143.9 (s), 142.2 (s), 140.1 (s), 128.3 (d), 128.1 (d), 127.8 (d), 127. 6 (d), 126.0 (d), 115.8 (t, C-5), 78.6 (d, C-1), 45.6 (t, C-3); HRMS: (EI, 70 eV) Calculated (C_17_H_15_I) 346.0218 (M^+^) Found: 346.0221.

*(Z)-(1-Iodo-3,3-dimethylpenta-1,4-dien-2-yl)benzene* (**4hd**)


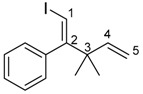


Phenylacetylene (1.02 mmol, 0.104 g) was added to a solution of InBr_3_ (1.01 mmol, 0.3597 g), prenyl trimethylsilane (1.96 mmol, 0.2788 g), and Me_2_Si(OMe)_2_ (0.962 mmol, 0.1157 g) in dichloromethane (1 mL). The mixture was stirred at room temperature for 24 h. The reaction mixture was cooled to −78 °C, and 0.75 M I_2_ in THF solution (2 mL) was added. The resultant mixture was stirred at −78 °C for 30 min. The mixture was quenched by saturated Na_2_S_2_O_3_ aq (10 mL). The mixture was extracted with dichloromethane (3 × 10 mL). The collected organic layer was dried over MgSO_4_. The solvent was evaporated and the residue was purified by column chromatography (hexane, column length 10 cm, diameter 26 mm silica gel) and GPC (CHCl_3_) to give the product (0.126 g, 41%).

IR: (neat) 1638, 1490, 1462, 1442 cm^−1^; ^1^H-NMR: (400 MHz, CDCl_3_) 7.41–7.7.32 (m, 3H, Ar), 7.04–7.00 (m, 2H, Ar), 6.53 (s, 1H, 1-H), 5.94 (dd, *J* = 17.4, 10.6 Hz, 1H, 4-H), 5.09 (d, *J* = 10.6 Hz, 1H, 5-H), 5.03 (d, *J* = 17.4 Hz, 1H, 5-H), 1.21 (s, 6H, 3-Me_2_); ^13^C-NMR: (100 MHz, CDCl_3_) 159.7 (s, C-2), 145.2 (d, C-4), 142. 4 (s), 128.9 (d), 127.8 (d), 127.1 (d), 112.3 (t, C-5), 80.2 (d, C-1), 45.1 (s, C-3), 26.4 (q, 3-Me_2_); HRMS: (EI, 70 eV) Calculated (C_13_H_15_I) 298.0218 (M^+^), Found: 298.0219.

*(Z)-1-Iodo-2,3-diphenylpenta-1,4-diene* (**4he**)


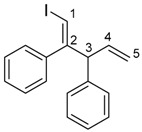


Phenylacetylene (1.02 mmol, 0.1044 g) was added to a solution of InBr_3_ (1.04 mmol, 0.3701 g), cinnamyl trimethylsilane (2.10 mmol, 0.4007 g), and Me_2_Si(OMe)_2_ (1.06 mmol, 0.1280 g) in dichloromethane (1 mL). The mixture was stirred at room temperature for 24 h. The reaction mixture was cooled to −78 °C, and 0.75 M I_2_ in THF solution (2 mL) was added. The resultant mixture was stirred at −78 °C for 30 min. The mixture was quenched by saturated Na_2_S_2_O_3_ aq (10 mL). The mixture was extracted with dichloromethane (3 × 10 mL). The collected organic layer was dried over MgSO_4_. The solvent was evaporated and the residue was purified by column chromatography (hexane, column length 10 cm, diameter 26 mm silica gel) and GPC (CHCl_3_) to give the product (0.080 g, 23%).

IR: (neat) 1636 cm^−1^; ^1^H-NMR: (400 MHz, CDCl_3_) 7.29–7.14 (m, 8H, Ar), 6.99 (dd, *J* = 7.8, 2.0 Hz, 2H, Ar), 6.42 (s, 1H, 1-H), 6.11 (ddd, *J* = 17.4, 10.1, 7.3 Hz, 1H, 4-H), 5.19 (d, *J* = 10.1 Hz, 1H, 5-H), 5.00 (d, *J* = 17.4 Hz, 1H, 5-H), 4.47 (d, *J* = 7.3 Hz, 1H, 3-H); ^13^C-NMR: (100 MHz, CDCl_3_) 154.1 (s), 142.3 (s), 139.8 (s), 138.2 (d, C-4), 128.48 (d), 128.40 (d), 128.2 (d), 127.9 (d), 127.4 (d), 126.8 (d), 117.3 (t, C-5), 80.4 (d, C-1), 58.8 (d, C-3); HRMS: (EI, 70 eV) Calculated (C_17_H_15_I) 346.0218 (M^+^), Found: 346.0214.

*(Z)-(4-Methyl-2-phenylpenta-1,4-dien-1-yl)indium(III) bromide* (**3ha**) 


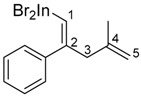


All manipulations were carried out in a globe box filled with nitrogen gas. Phenylacetylene (0.886 mmol, 0.0905 g) was added to a solution of InBr_3_ (1.00 mmol, 0.3550 g), methallyl trimethylsilane (1.98 mmol, 0.2541 g), and Me_2_Si(OMe)_2_ (1.05 mmol, 0.1267 g) in dichloromethane (1 mL). The mixture was stirred at room temperature for 24 h. The volatiles were evaporated and the residual oil was washed with hexane to obtain the desired alkenylindium compound as a white solid (0.106 g, 26%).

^1^H-NMR: (400 MHz, CDCl_3_) 7.43–7.22 (m, 5H, Ar), 5.99 (s, 1H, 1-H), 4.83 (s, 1H, 5-H), 4.73 (s, 1H, 5-H), 3.30 (s, 2H, 3-H_2_), 1.72 (s, 3H, 4-Me); ^13^C-NMR: (100 MHz, CDCl_3_) 160.6 (s), 145.7 (s), 141.9 (s), 134.1 (d, C-1), 129.5 (d), 128.8 (d), 126.5 (d), 113.9 (t, C-5), 48.1 (t, C-3), 22.1 (q, 4-Me).

*(Z)-4-Methy-1,2-diphenylpenta-1,4-diene* (**5**)


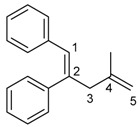


Phenylacetylene (0.540 mmol, 0.0551 g) was added to a solutin of InBr_3_ (0.532 mmol, 0.1885 g), methallyl trimethylsilane (1.01 mmol, 0.1290 g), and Me_2_Si(OMe)_2_ (0.499 mmol, 0.060 g) in dichloromethane (0.5 mL). The mixture was stirred at room temperature for 3 h. DMF (1 mL) was added to the reaction mixture at −78 °C. Then, the »reaction mixture was warmed to room temperature. PhI (0.749 mmol, 0.1528 g) and Pd(PPh_3_)_4_ (0.028 mmol, 0.0325g) were added to the reaction mixture, and the mixture was heated at 100 °C for 3 h. The mixture was quenched by H_2_O (10 mL) and Et_2_O (20 mL) at room temperature. The organic layer was washed by H_2_O (3 × 10 mL), and was dried over MgSO_4_. The solvent was evaporated and the residue was purified by column chromatography (hexane, column length 10 cm, diameter 26 mm silica gel) and GPC (CHCl_3_) to give the product (0.0686 g, 54%).

IR: (neat) 1650, 1599, 1494, 1444 cm^−1^; ^1^H-NMR: (400 MHz, CDCl_3_) 7.29–7.20 (m, 3H, Ar), 7.15 (d, *J* = 6.8 Hz, 2H, Ar), 7.12–7.04 (m, 3H, Ar), 6.95 (d, *J* = 6.8 Hz, Ar), 4.79 (s, 1H, 5-H), 4.72 (s, 1H, 5-H), 3.18 (s, 2H, 3-H_2_), 1.76 (s, 3H, 4-Me); ^13^C-NMR: (100 MHz, CDCl_3_) 142.9 (s, C-4), 141.1 (s), 140.4 (s), 137.3 (s), 129.0 (d), 128.6 (d), 128.3 (d), 128.1 (d), 127.8 (d), 126.9 (d), 126.3 (d), 113.1 (t, C-5), 49.1 (t, C-3), 22.1 (q, 4-Me); HRMS: (EI, 70 eV) Calculated (C_18_H_18_) 234.1409 (M^+^), Found: 234.1408.

## 4. Conclusions

We established a regioselective *anti*-allylindation of alkynes using InBr_3_ and allylic silanes. Many types of aliphatic and aromatic alkynes were applicable. The present allylindation has a wide scope of allylic silanes, and the reactions using allyl, methallyl, prenyl, cinnamyl silanes gave the desired products. A 1,4-dienyl indium compound generated by the present allylindation was successfully isolated and characterized by NMR spectroscopy. The synthesized 1,4-dienyl indiums were applicable to iodination and Pd-catalyzed cross-coupling with an aryl iodide in a one-pot manner to give the corresponding functionalized skipped dienes.

## Figures and Tables

**Table 1:**
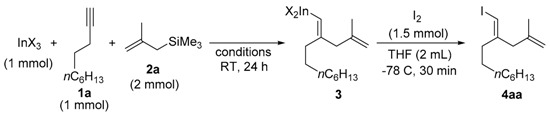
Optimization of reaction conditions for carboindation of alkyne **1a** with allylic silane **2a** ^a^.

Entry	InX_3_	Solvent	Yield/%
1	InBr_3_	CH_2_Cl_2_	89
2 ^b^	InCl_3_	CH_2_Cl_2_	42
3	InF_3_	CH_2_Cl_2_	0
4	InI_3_	CH_2_Cl_2_	0
5	In(OTf)_3_	CH_2_Cl_2_	0
6	InBr_3_	toluene	0
7	InBr_3_	Et_2_O	0
8	InBr_3_	CH_3_CN	0
9	InBr_3_	THF	0

^a^ InX_3_ (1 mmol), alkyne **1a** (1 mmol), allylic silane **2a** (2 mmol), solvent (1 mL), room temperature, 24 h. I_2_ (1.5 mmol), THF (2 mL). Yields were determined via ^1^H-NMR using 1,1,2,2-tetrachloroethane as an internal standard; ^b^ Et_2_O was used instead of THF.

**Table 2:**
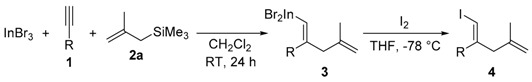
Scope and limitation of alkyne **1** in allylindation ^a^.

Entry	Alkyne 1	Product 4	Yield/%
1	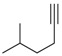 **1b**	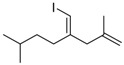 **4ba**	64
2	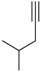 **1c**	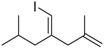 **4ca**	41
3	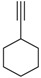 **1d**	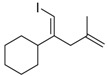 **4da**	40
4	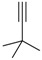 **1e**	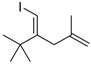 **4ea**	0
5	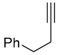 **1f**	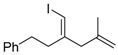 **4fa**	59
6	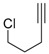 **1g**	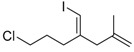 **4ga**	80
7	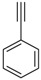 **1h**	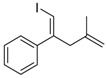 **4ha**	65
8			78 ^b^

^a^ Alkyne **1** (1 mmol), allylic silane **2a** (2 mmol), InBr_3_ (1 mmol), CH_2_Cl_2_ (1 mL). Yields were determined by ^1^H-NMR using 1,1,2,2-tetrachloroethane as an internal standard; ^b^ Me_2_Si(OMe)_2_ (1 mmol) was added.

**Table 3:**
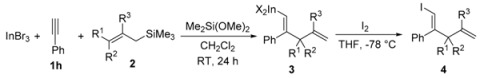
Scope of allylic silane **2** in allylindation ^a^.

Entry	Allylic Silane 2	Product 3	Yield/%
1	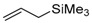 **2b**	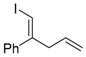 **4hb**	48
2	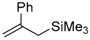 **2c**	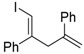 **4hc**	76
3	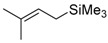 **2d**	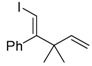 **4hd**	72
4	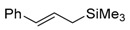 **2e**	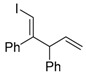 **4he**	39

^a^ Alkyne **1a** (1 mmol), allylic silane **2** (2 mmol), InBr_3_ (1 mmol), Me_2_Si(OMe)_2_ (1 mmol), and CH_2_Cl_2_ (1 mL). Yields were determined by ^1^H-NMR using 1,1,2,2-tetrachloroethane as an internal standard.

## Supplementary Materials

The following are available online, Supporting Information of NOE Experiments and NMR Spectra.
