# Supplementary material for: Regio- and Stereoselective Allylindation of Alkynes Using InBr3 and Allylic Silanes: Synthesis, Characterization, and Application of 1,4-Dienylindiums toward Skipped Dienes
Source: Molecules. 2018 Jul 27;23(8):1884. doi: 10.3390/molecules23081884 (PMC6222668; doi:10.3390/molecules23081884)

Supporting Information

Regio- and Stereoselective Allylindation of Alkynes Using $InBr_3$ and Allylic Silanes: Synthesis, Characterization, and Application of 1,4-Dienylindiums toward Skipped Dienes

Yoshihiro Nishimoto[1,*], Yi Junyi[2], Tatsuaki Takata[2], Akio Baba[2], and Makoto Yasuda[2,*]

[1]Frontier Research Base for Global Young Researchers Center for Open Innovation Research and Education (COiRE), Graduate School of Engineering, Osaka University

[2]Department of Applied Chemistry, Graduate School of Engineering, Osaka University

Contents

NOE Experiments **2**

NMR Spectra **4**

NOE Experiments

1,4-dienylindium 3ha

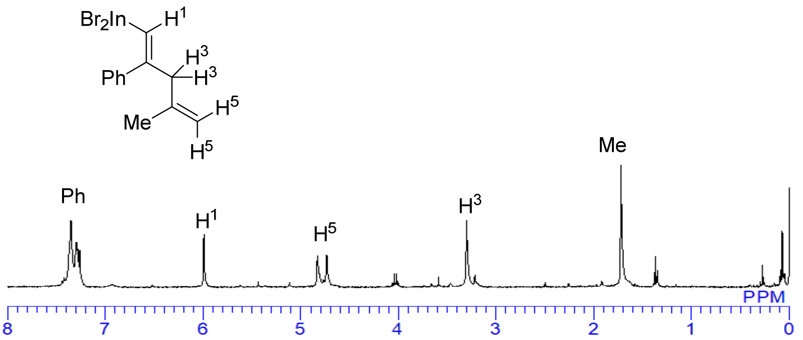

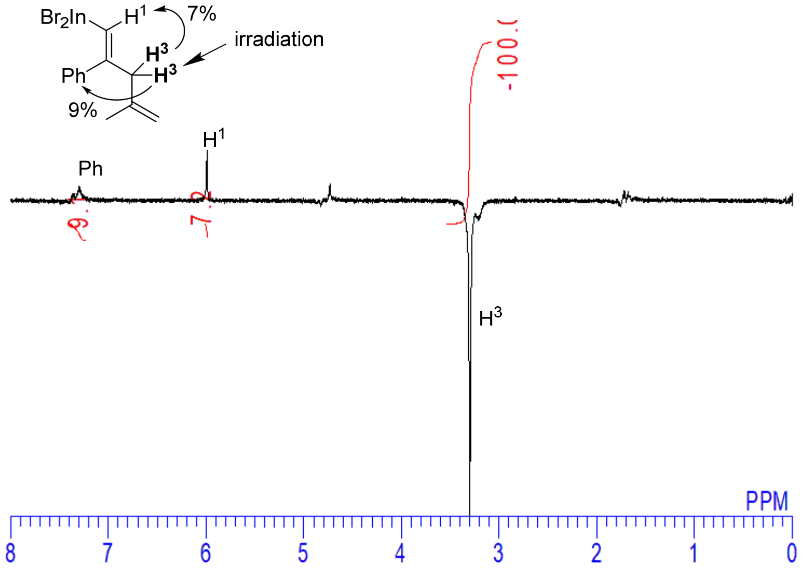

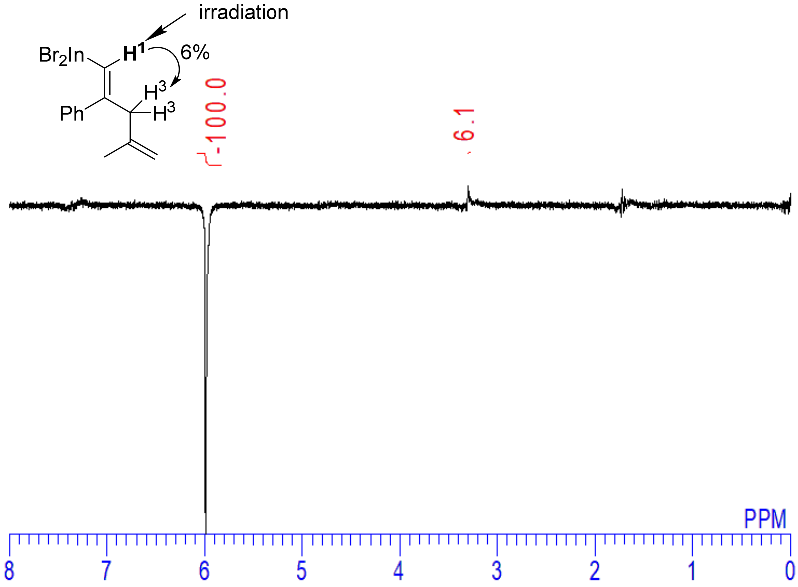

(Z)-(1-iodo-4-methylpenta-1,4-dien-2-yl)benzene (4ha)

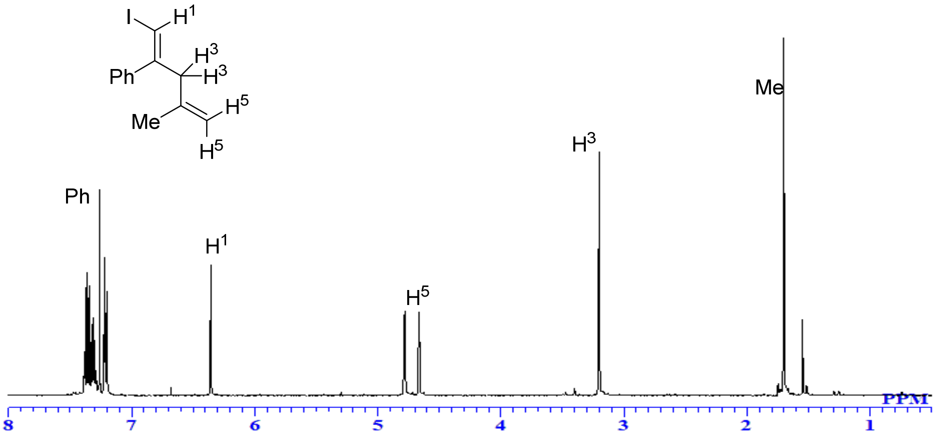

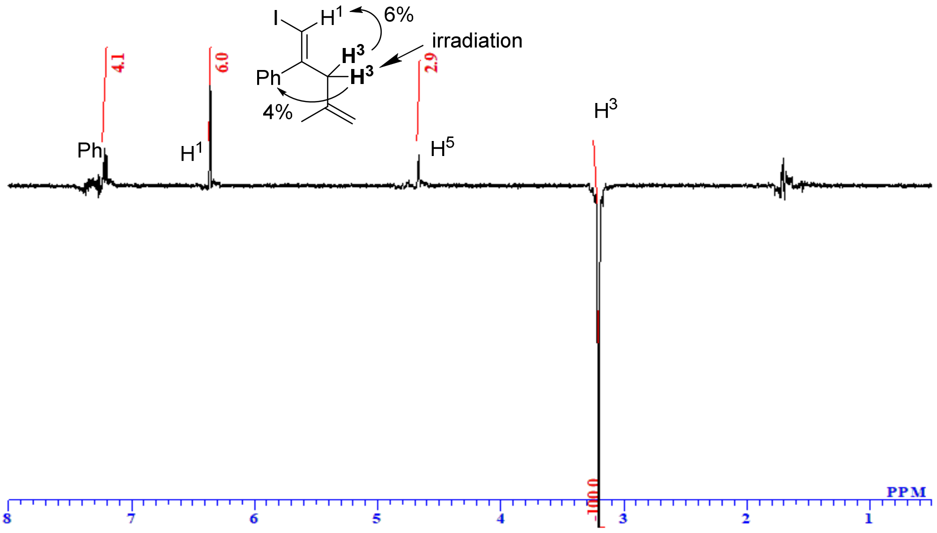

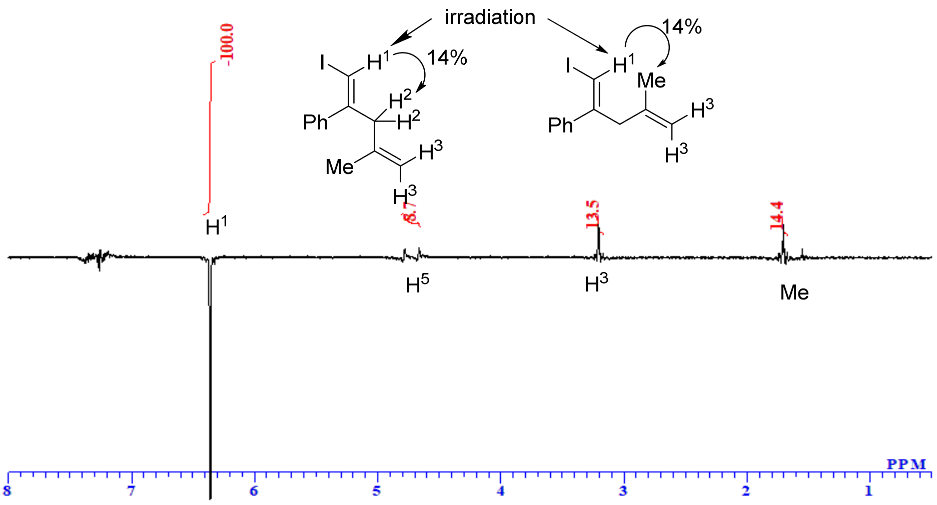

NMR Spectra

(E)-4-(iodomethylene)-2-methyldodec-1-ene (**4aa**)

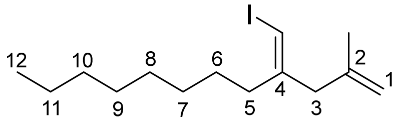

^{1}H NMR (400 MHz, in $CDCl_3$)

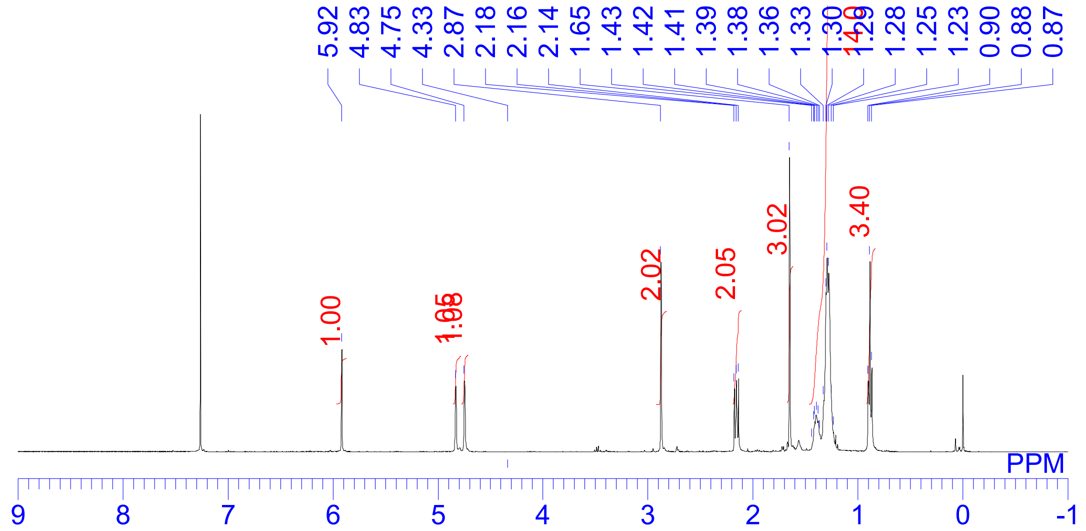

^{13}C NMR (100 MHz, in $CDCl_3$)

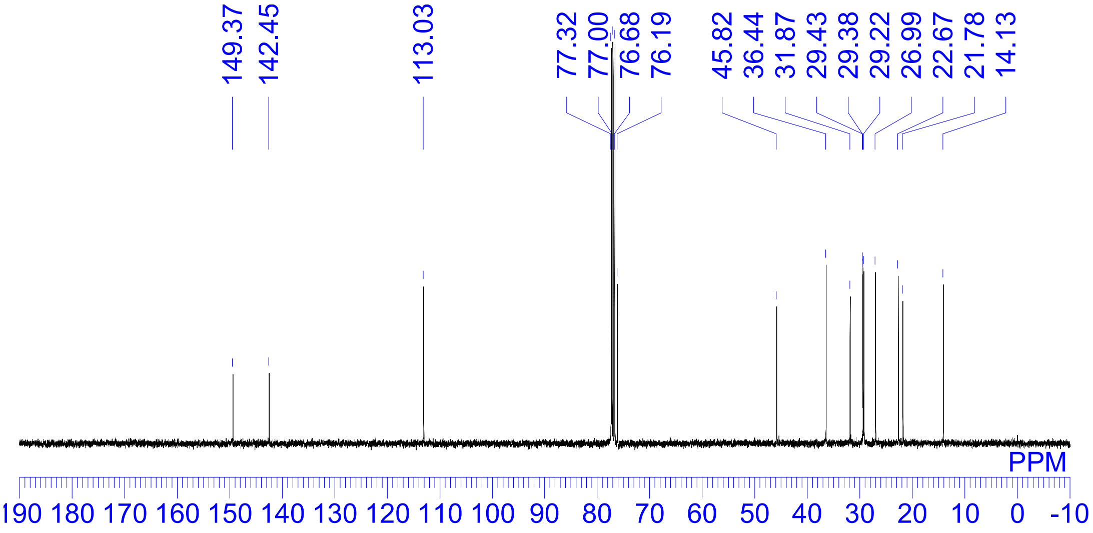

HMQC

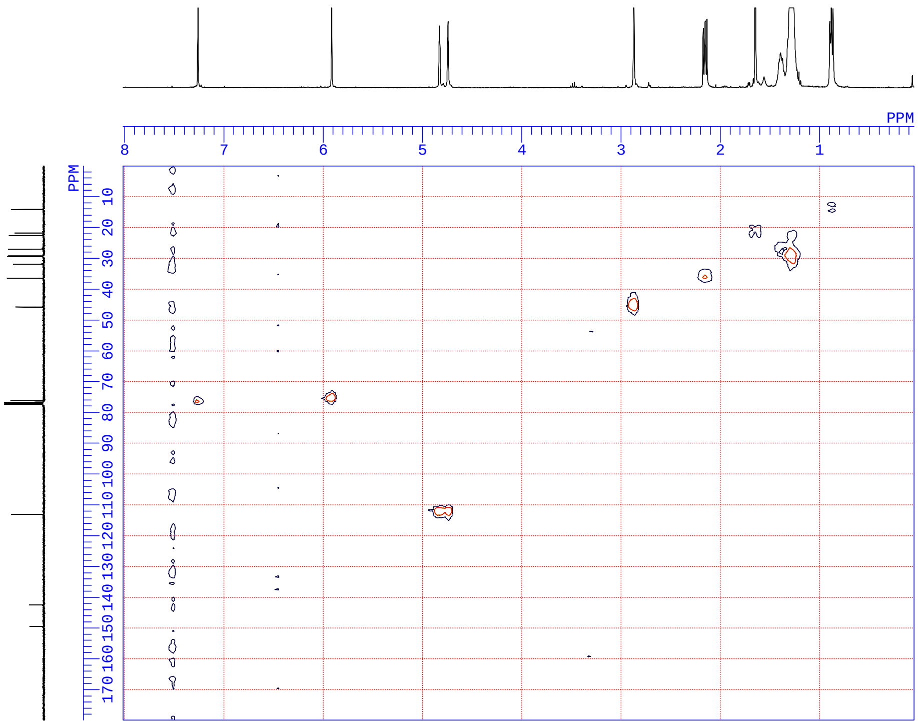
PPM
8
7
6
5
4
3
2
1
PPM
10
20
30
40
50
60
70
80
90
100
110
120
130
140
150
160
170

HMBC

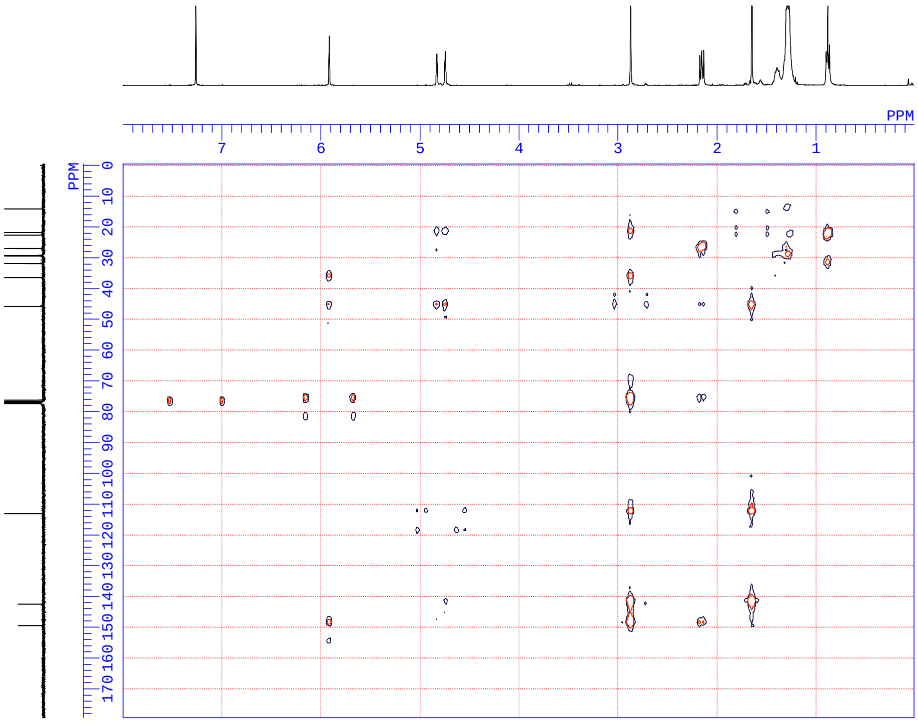
PPM
7
6
5
4
3
2
1
PPM
0
10
20
30
40
50
60
70
80
90
100
110
120
130
140
150
160
170

(E)-4-(iodomethylene)-2,7-dimethyloct-1-ene (**4ba**)

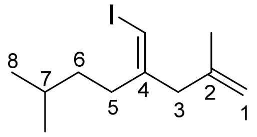

^{1}H NMR (400 MHz, in $CDCl_3$)

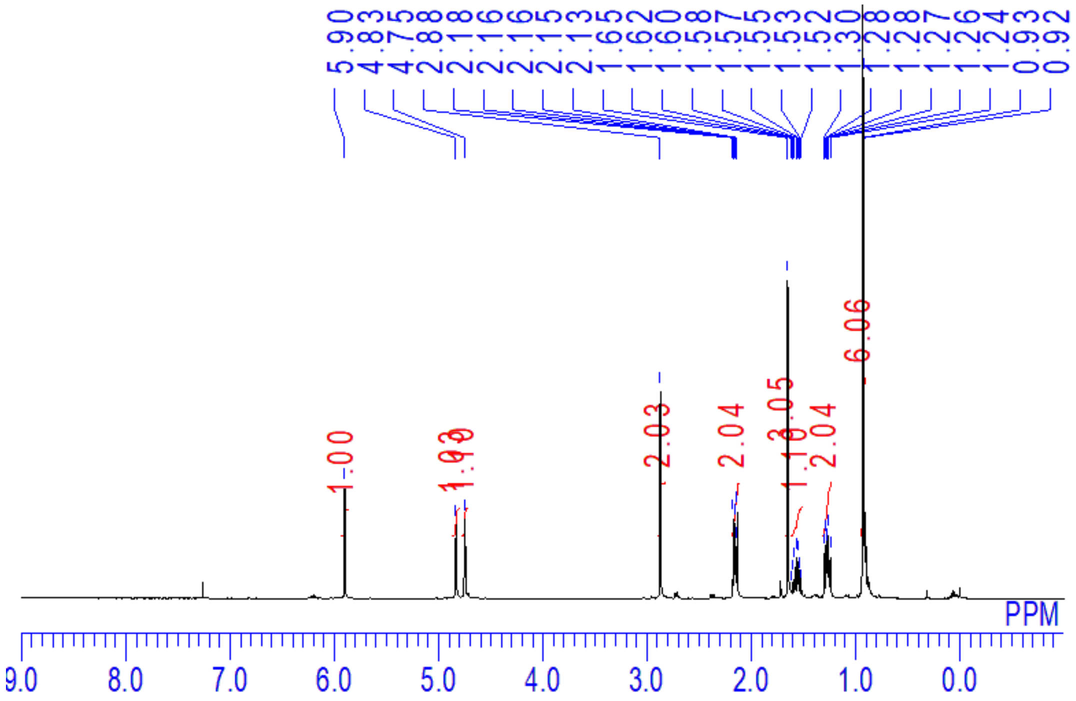

^{13}C NMR (100 MHz, in $CDCl_3$)

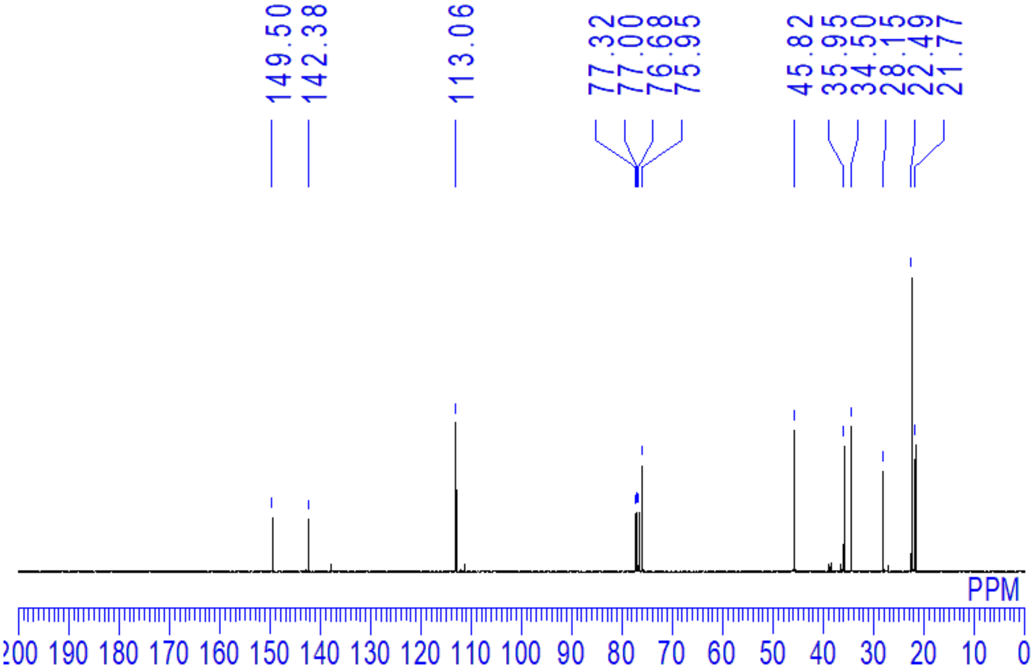

(E)-4-(iodomethylene)-2,6-dimethylhept-1-ene (**4ca**)

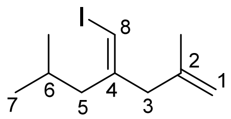

^{1}H NMR (400 MHz, in $CDCl_3$)

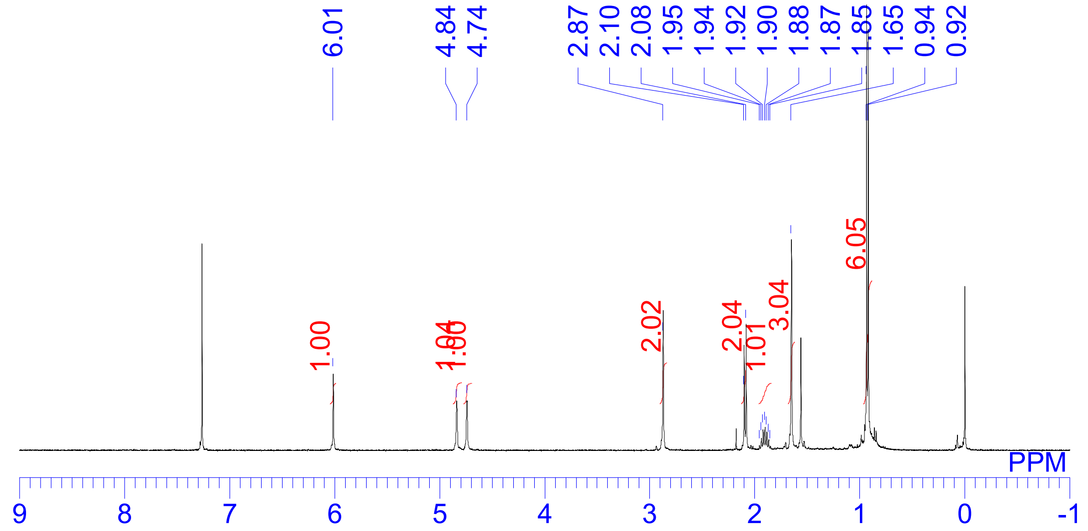

^{13}C NMR (100 MHz, in $CDCl_3$)

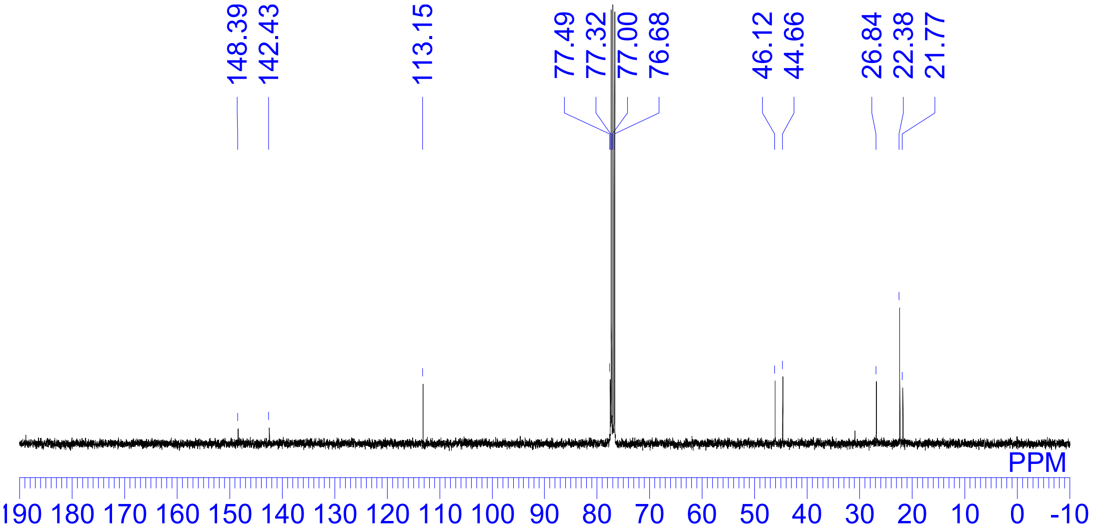

(Z)-(1-iodo-4-methylpenta-1,4-dien-2-yl)cyclohexane (**4da**)

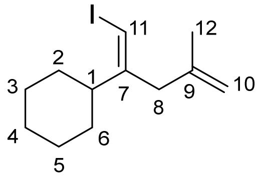

^{1}H NMR (400 MHz, in $CDCl_3$)

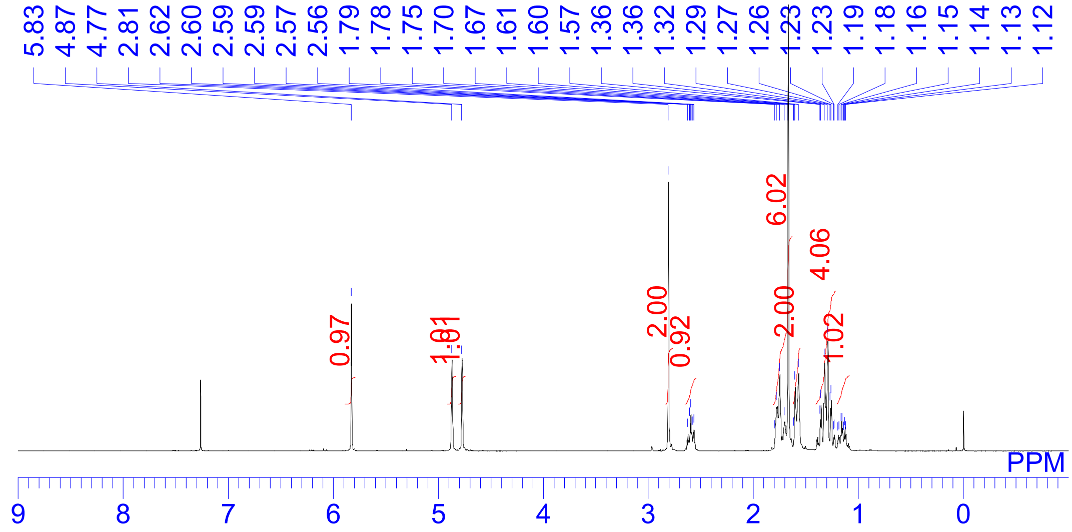

^{13}C NMR (400 MHz, in $CDCl_3$)

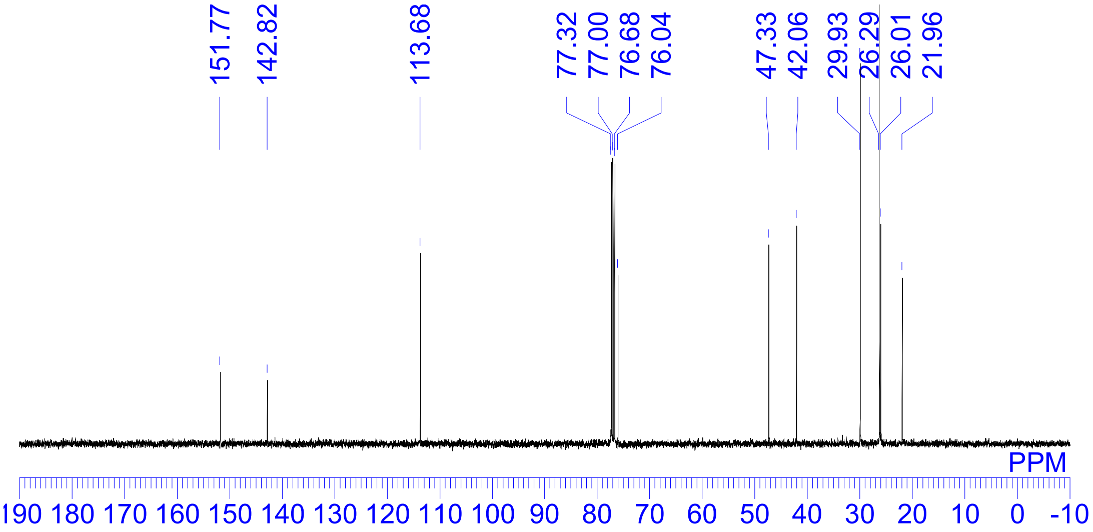

(E)-(3-(iodomethylene)-5-methylhex-5-en-1-yl)benzene (**4fa**)

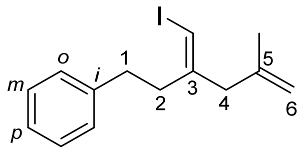

^{1}H NMR (400 MHz, in $CDCl_3$)

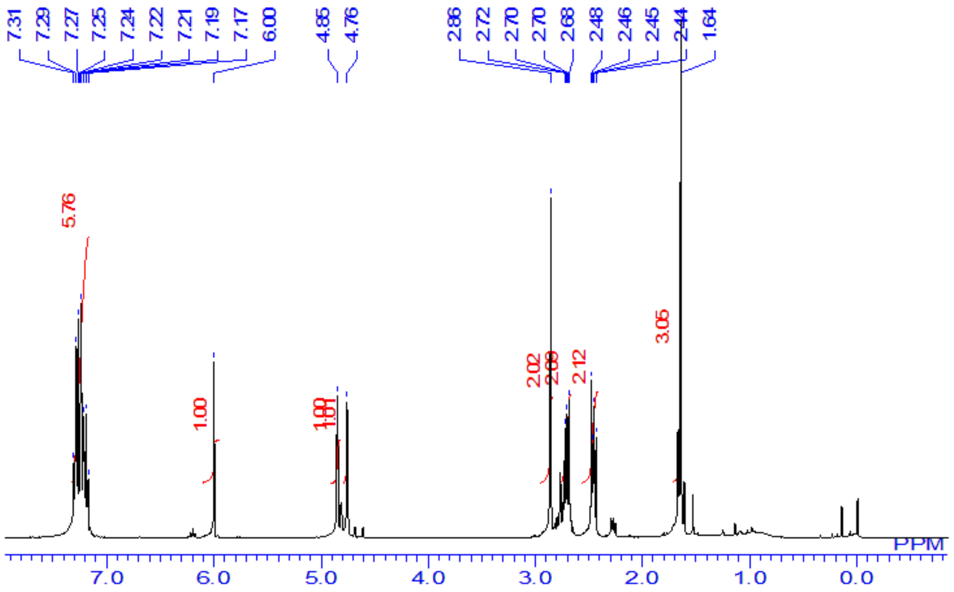

^{13}C NMR (100 MHz, in $CDCl_3$)

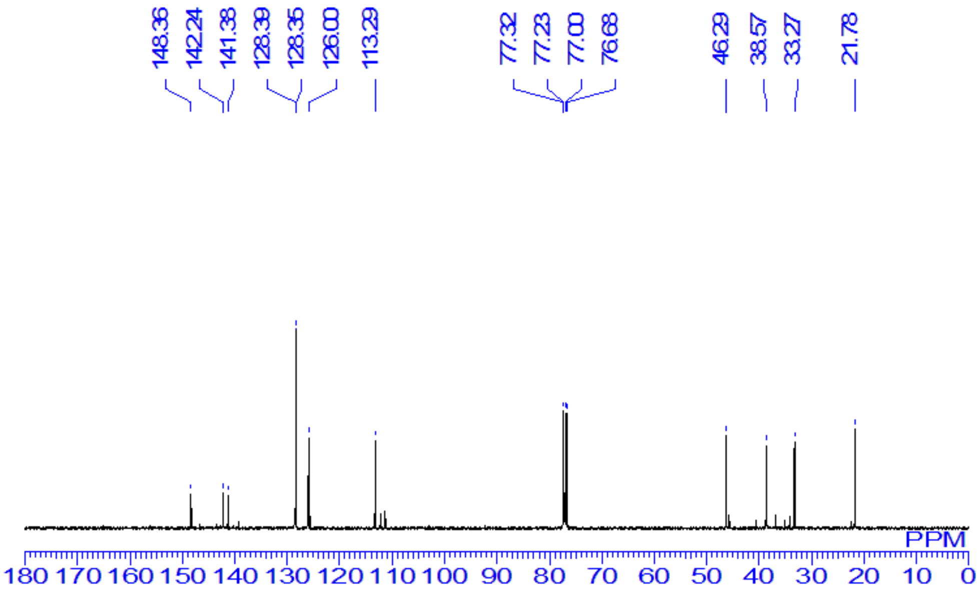

(E)-7-chloro-4-(iodomethylene)-2-methylhept-1-ene (**4ga**)

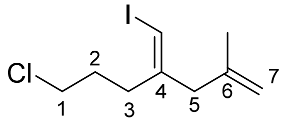

^{1}H NMR (400 MHz, $CDCl_3$)

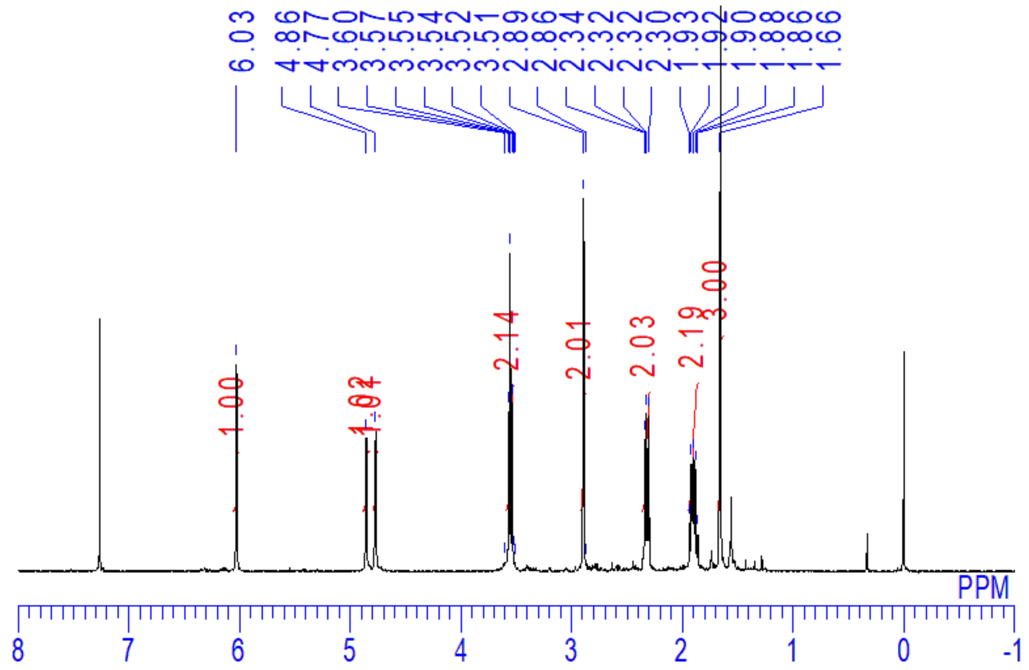

^{13}C NMR (100 MHz, $CDCl_3$)

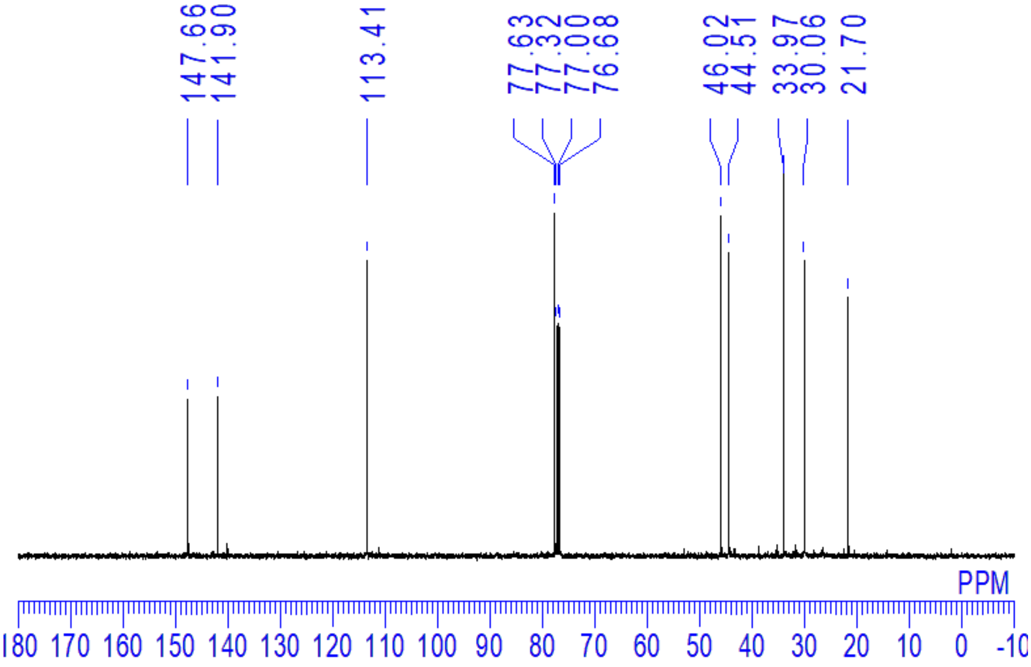

HMQC

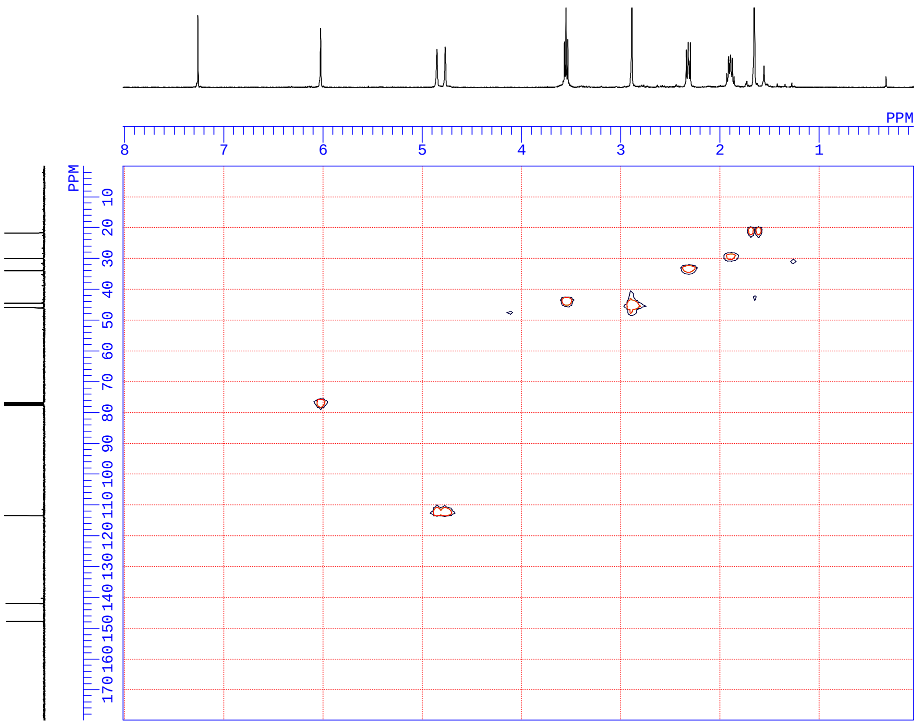
PPM
8
7
6
5
4
3
2
1
PPM
10
20
30
40
50
60
70
80
90
100
110
120
130
140
150
160
170

HMBC

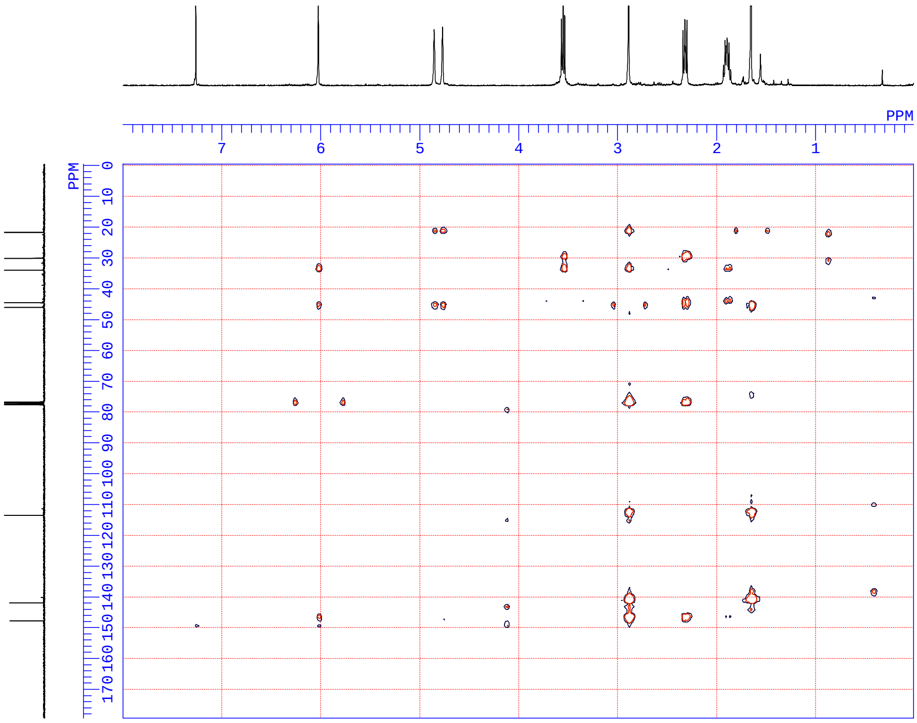
PPM
7
6
5
4
3
2
1
PPM
0
10
20
30
40
50
60
70
80
90
100
110
120
130
140
150
160
170

(Z)-(1-iodo-4-methylpenta-1,4-dien-2-yl)benzene (**4ha**)

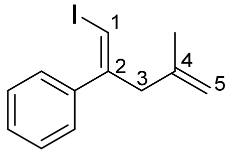

^{1}H NMR (400 MHz, in $CDCl_3$)

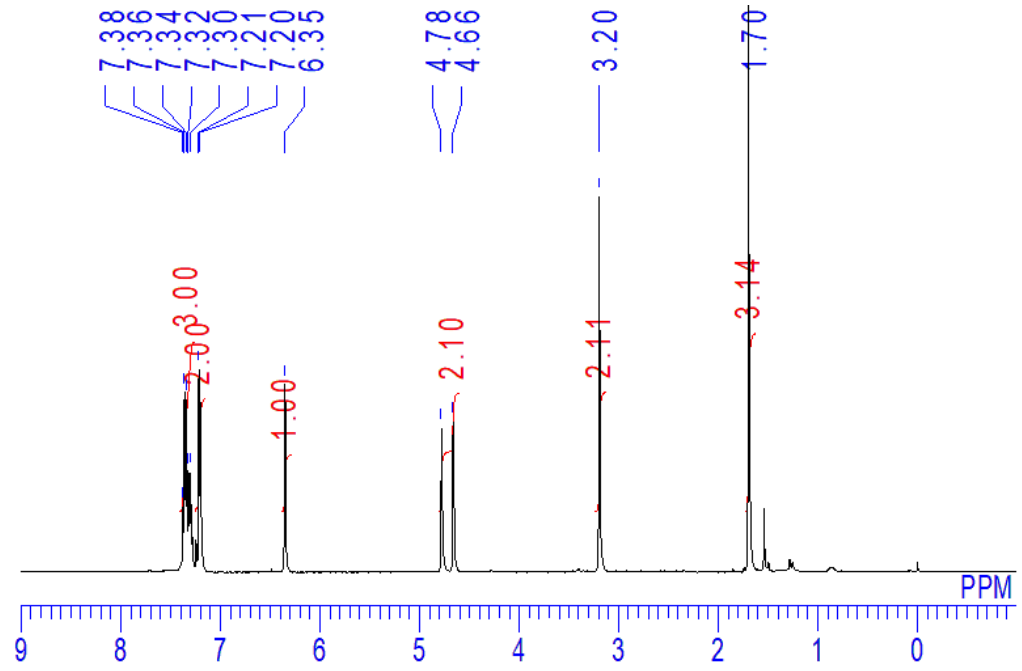

^{13}C NMR (100 MHz, in $CDCl_3$)

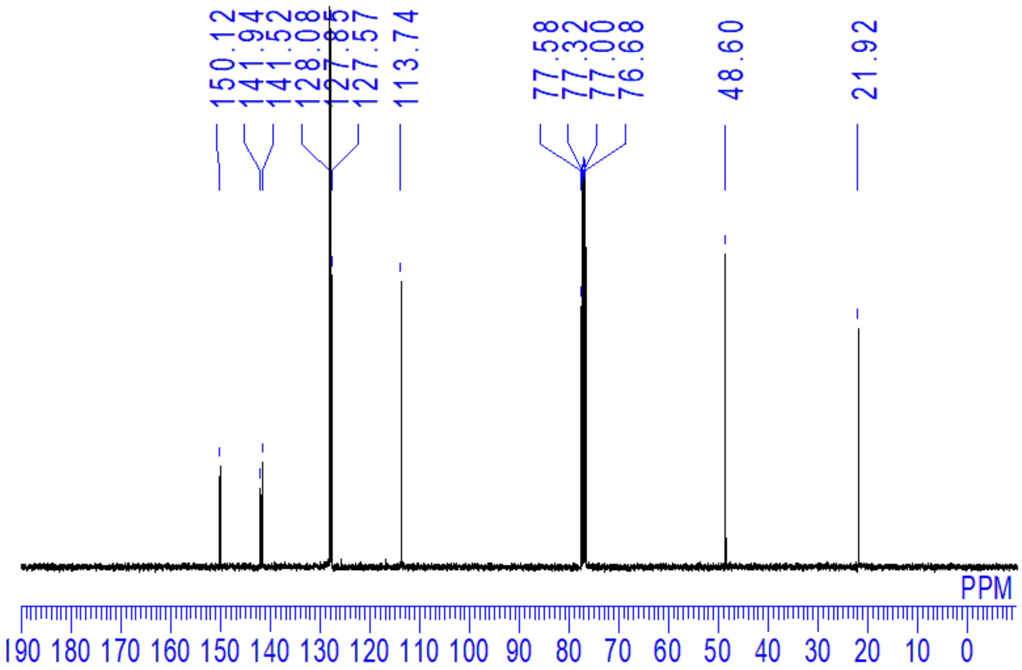

HMQC

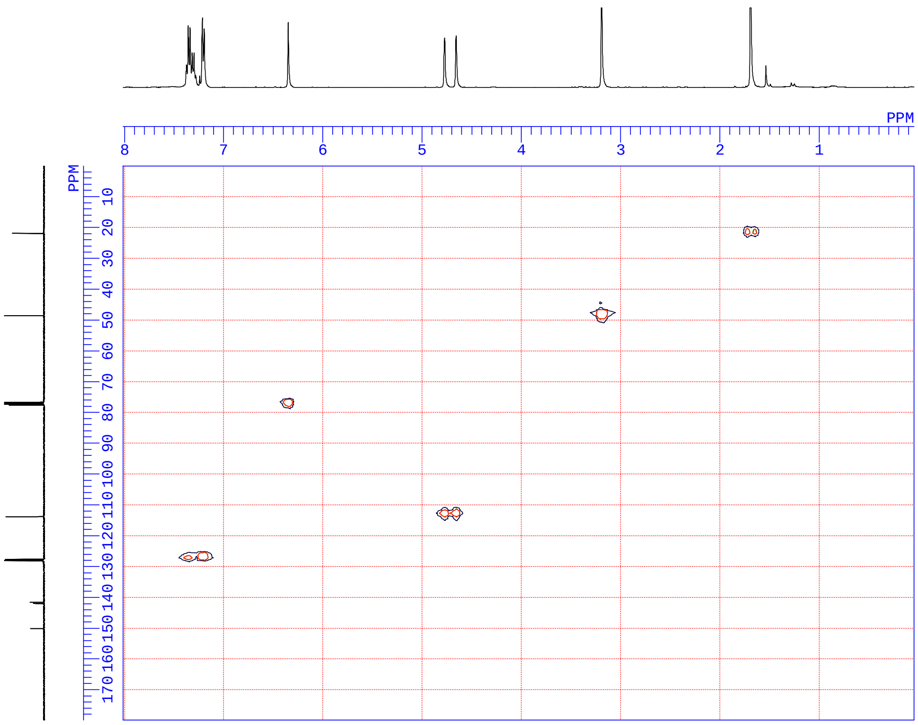
PPM
8
7
6
5
4
3
2
1
PPM
10
20
30
40
50
60
70
80
90
100
110
120
130
140
150
160
170

HMBC

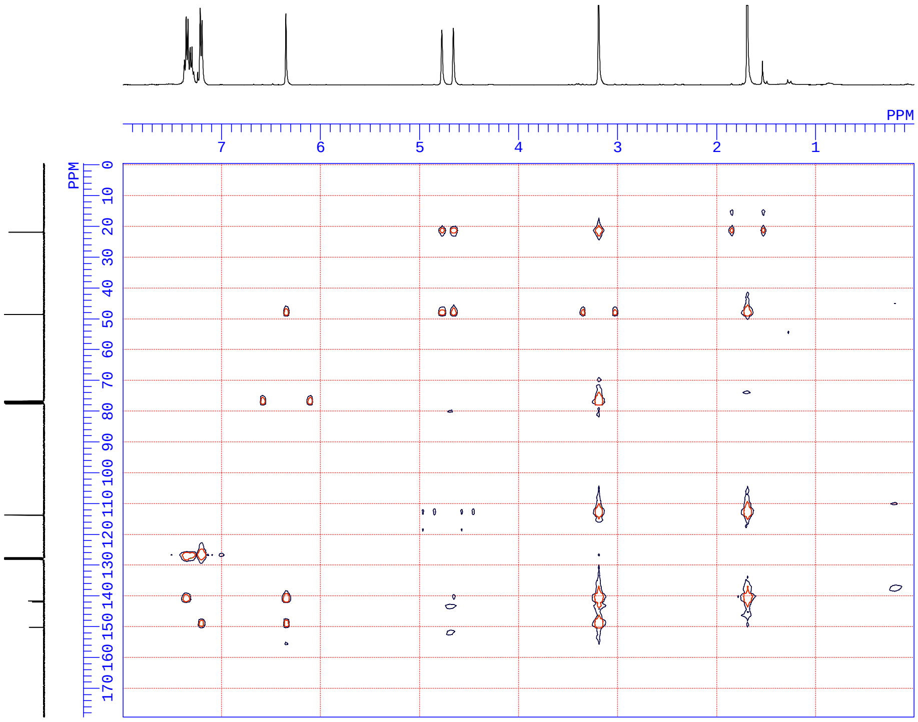
PPM
7
6
5
4
3
2
1
PPM
0
10
20
30
40
50
60
70
80
90
100
110
120
130
140
150
160
170

(Z)-(1-iodopenta-1,4-dien-2-yl)benzene (**4hb**)

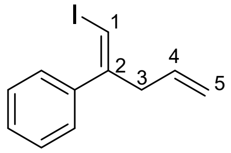

^{1}H NMR (400 MHz, in $CDCl_3$)

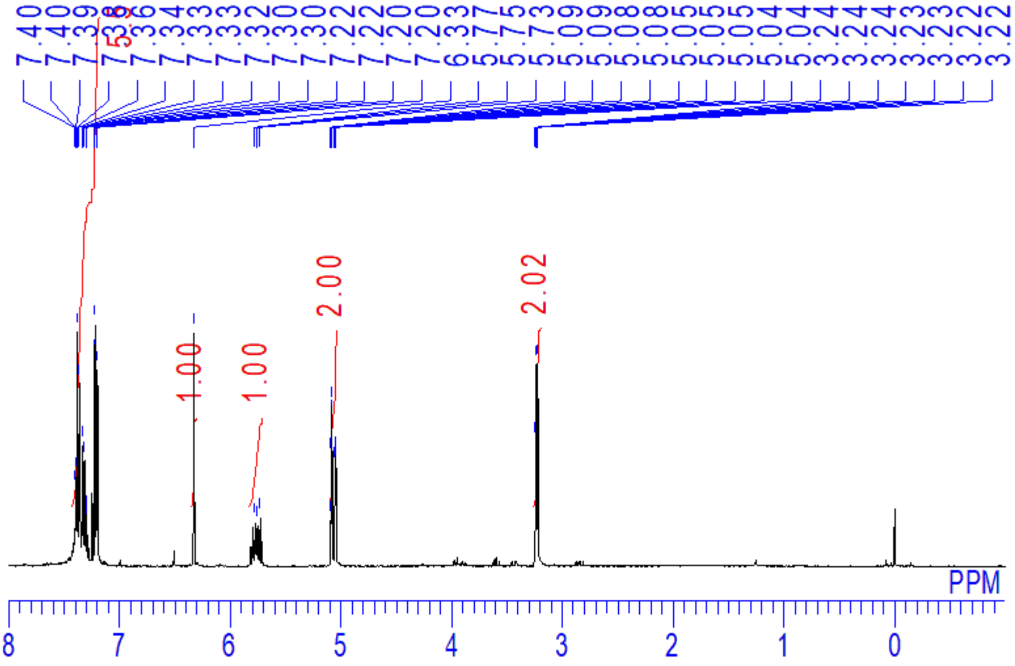

^{13}C NMR (100 MHz, in $CDCl_3$)

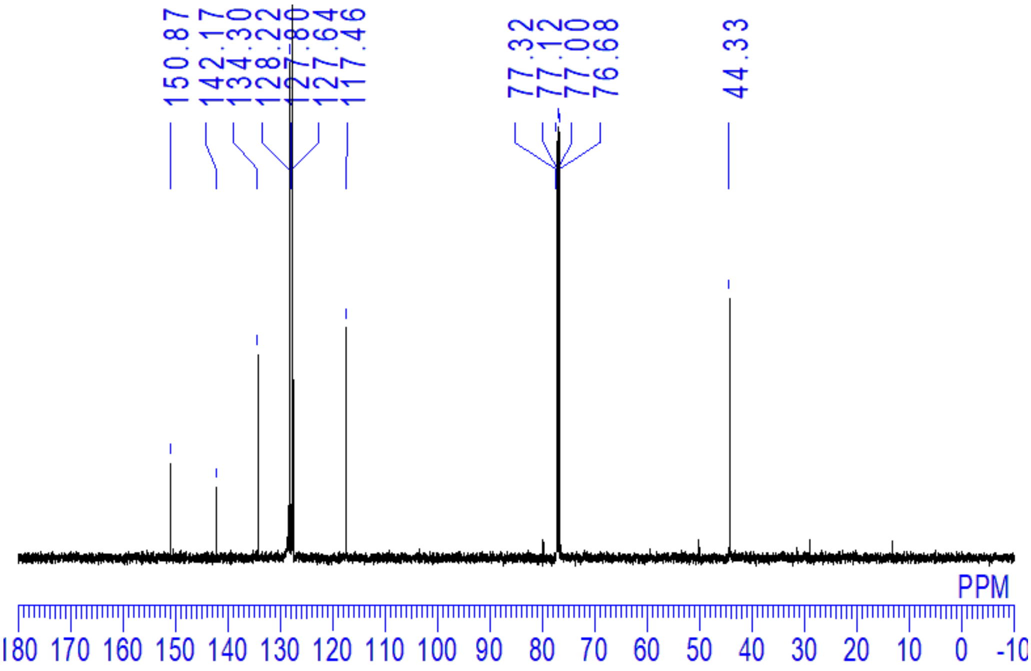

(Z)-1-iodo-2,4-diphenylpenta-1,4-diene (**4hc**)

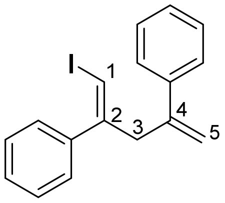

^{1}H NMR (400 MHz, in $CDCl_3$)

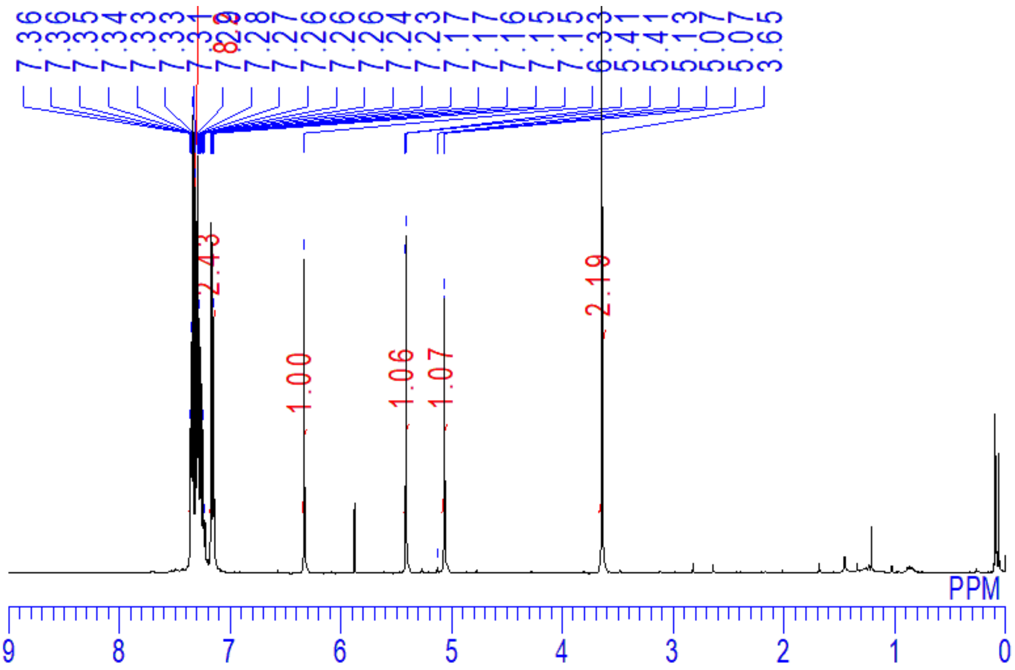

^{13}C NMR (100 MHz, in $CDCl_3$)

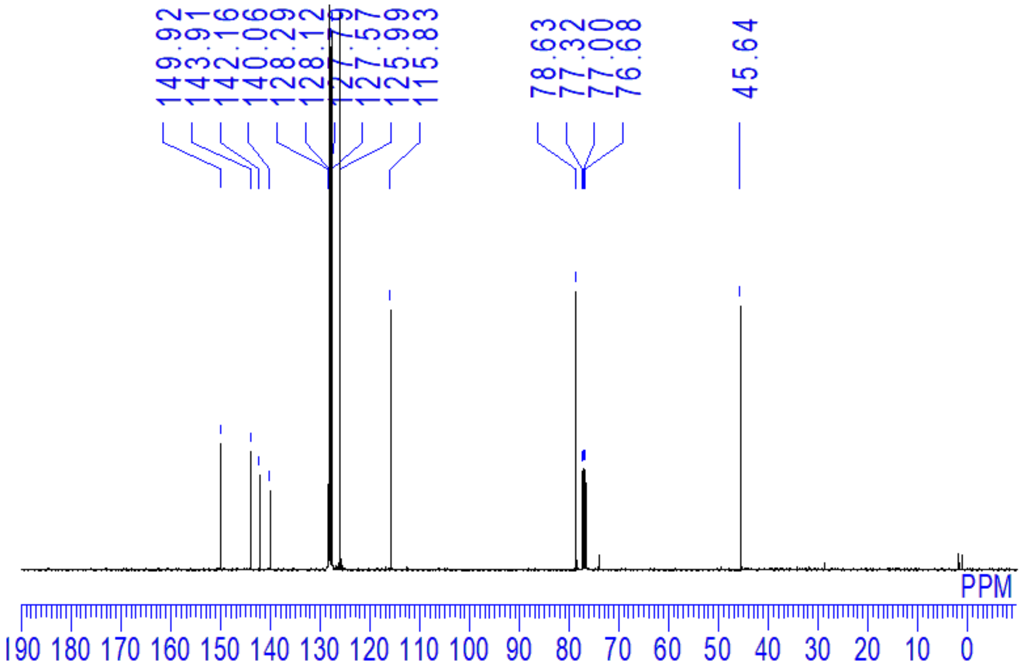

(Z)-(1-iodo-3,3-dimethylpenta-1,4-dien-2-yl)benzene (**4hd**)

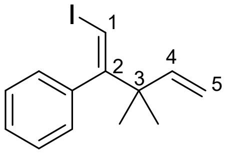

^{1}H NMR (400 MHz, $CDCl_3$)

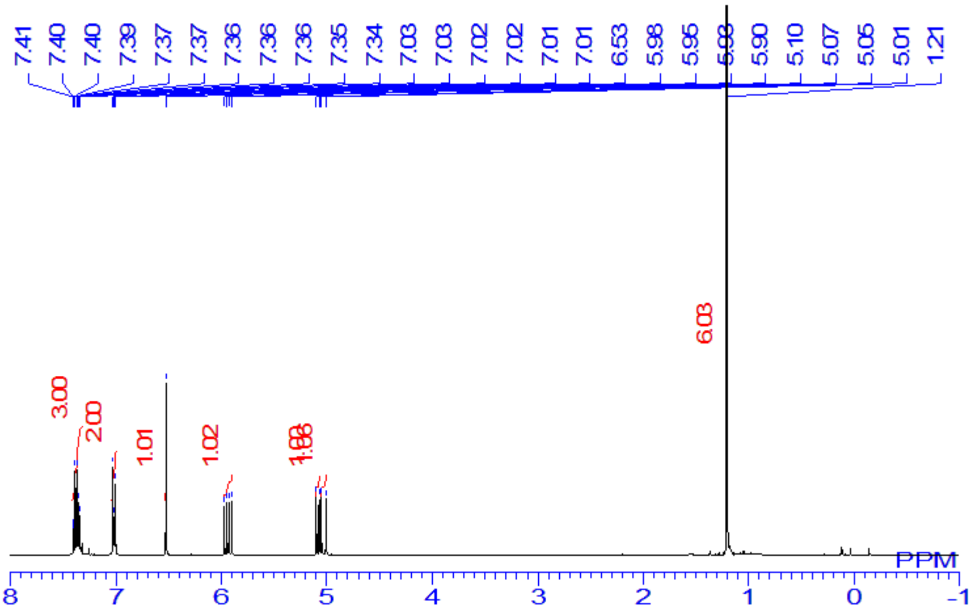

^{13}C NMR (100 MHz, $CDCl_3$)

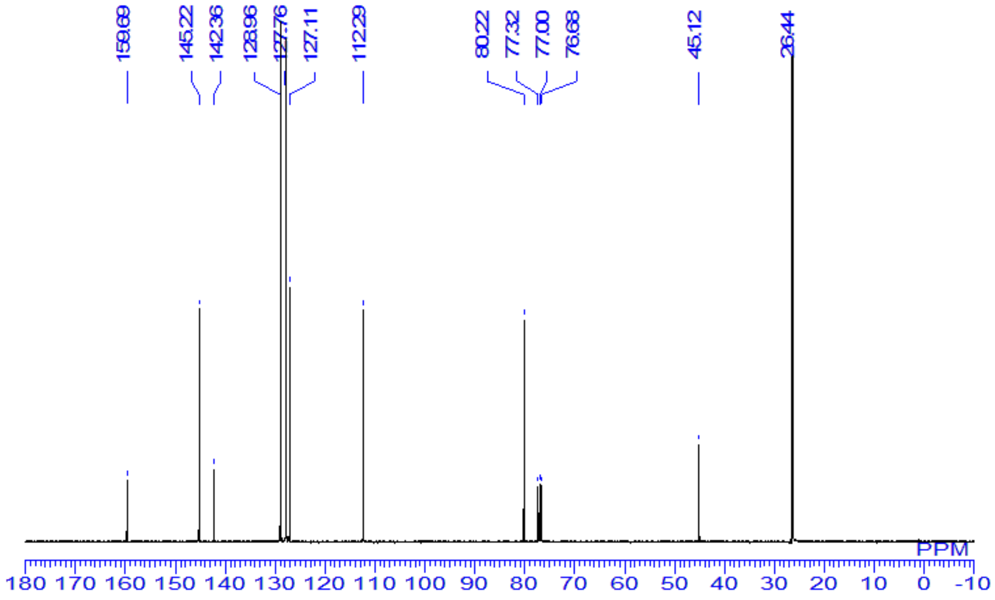

(Z)-1-iodo-2,3-diphenylpenta-1,4-diene (**4he**)

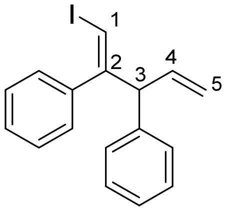

^{1}H NMR (400 MHz, in $CDCl_3$)

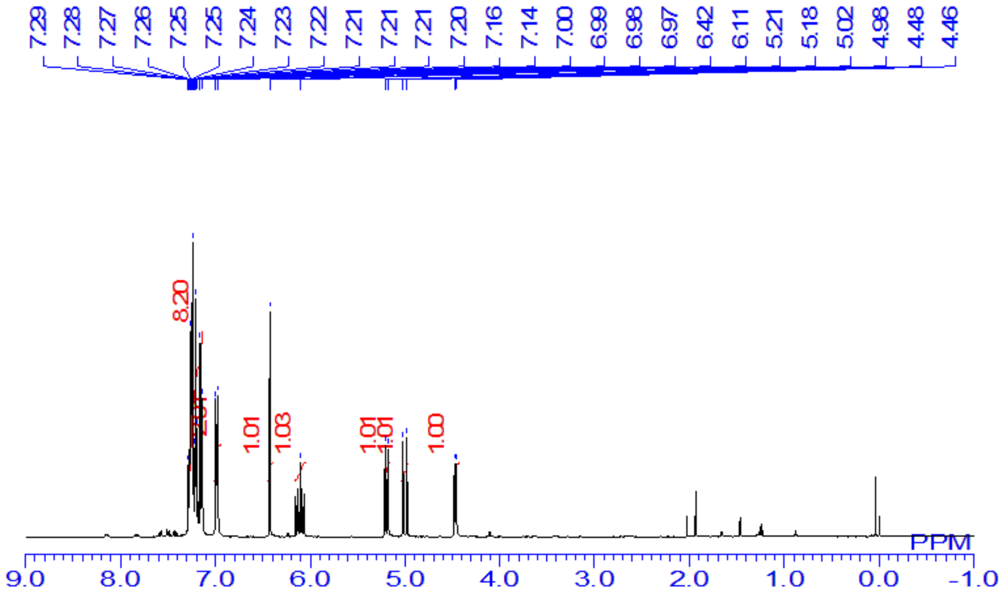

^{13}C NMR (100 MHz, in $CDCl_3$)

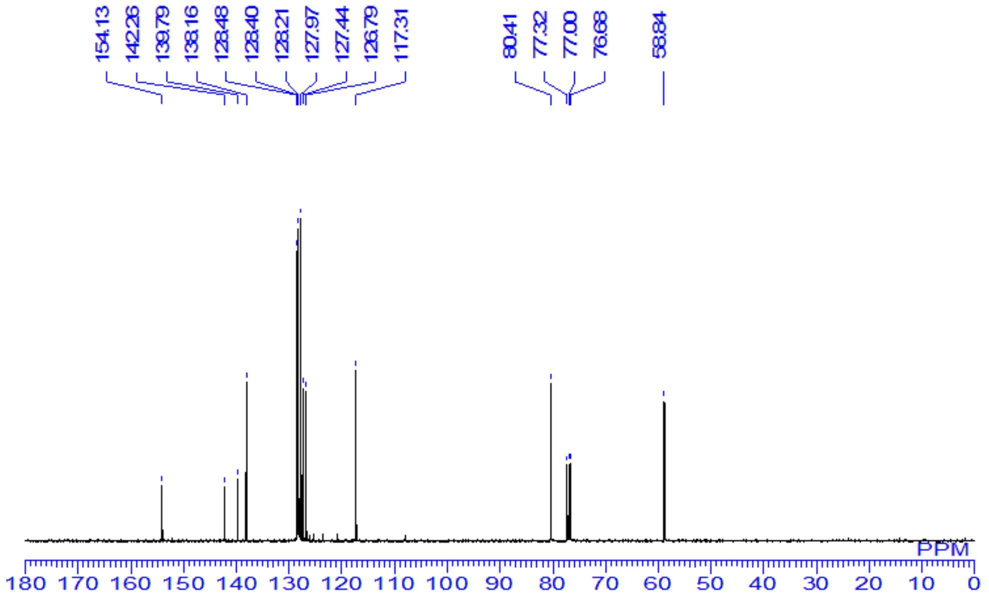

(Z)-(4-methyl-2-phenylpenta-1,4-dien-1-yl)indium(III) bromide (**3ha**)

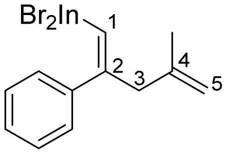

^{1}H NMR (400 MHz, in $CDCl_3$)

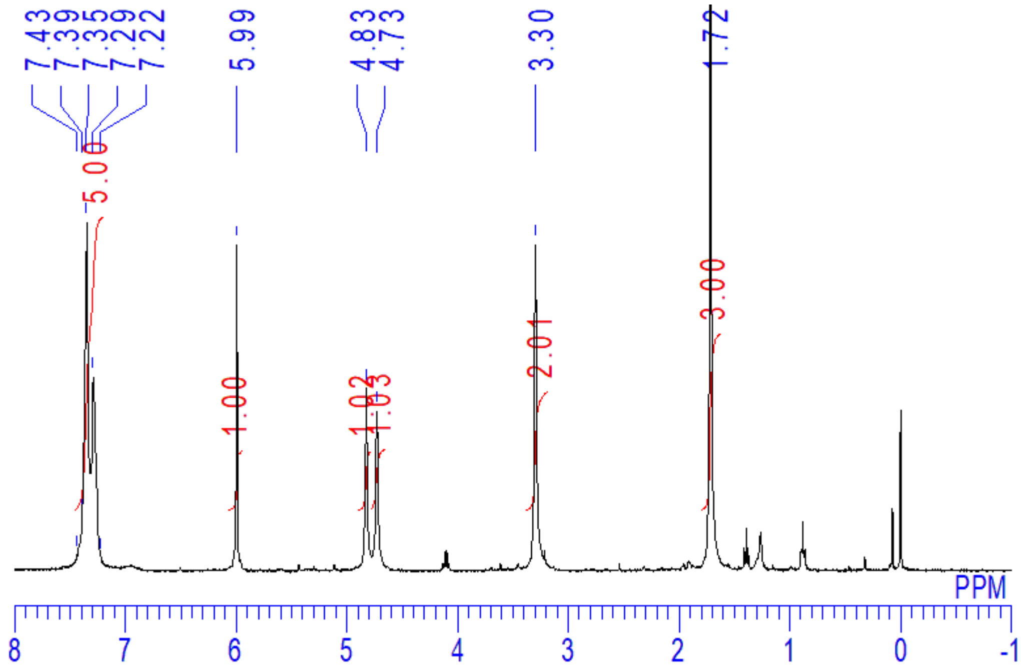

^{13}C NMR (100 MHz, in $CDCl_3$)

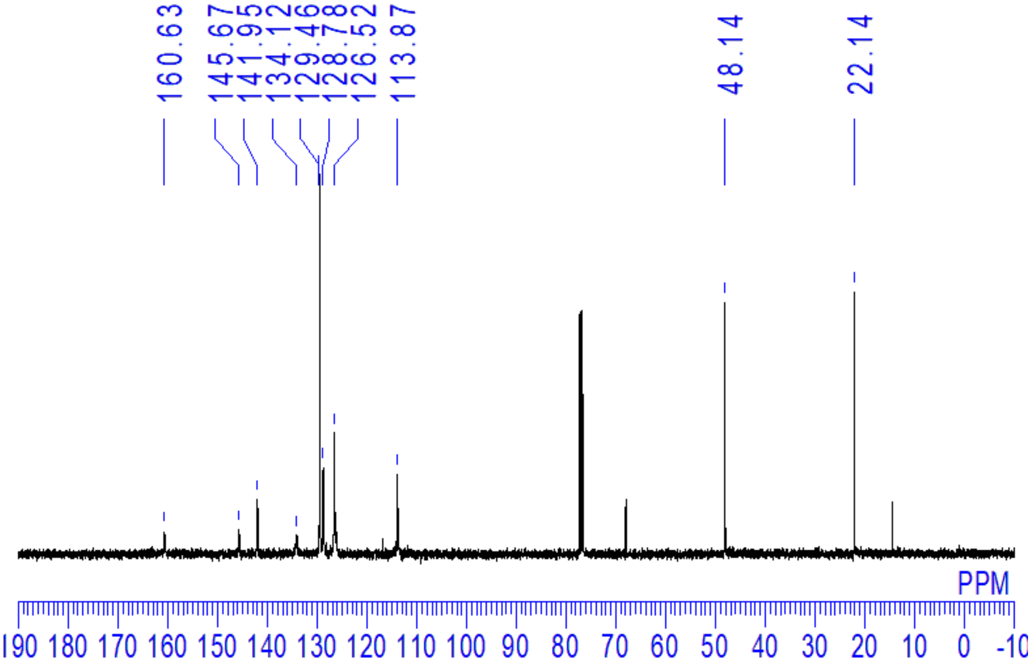

HMQC

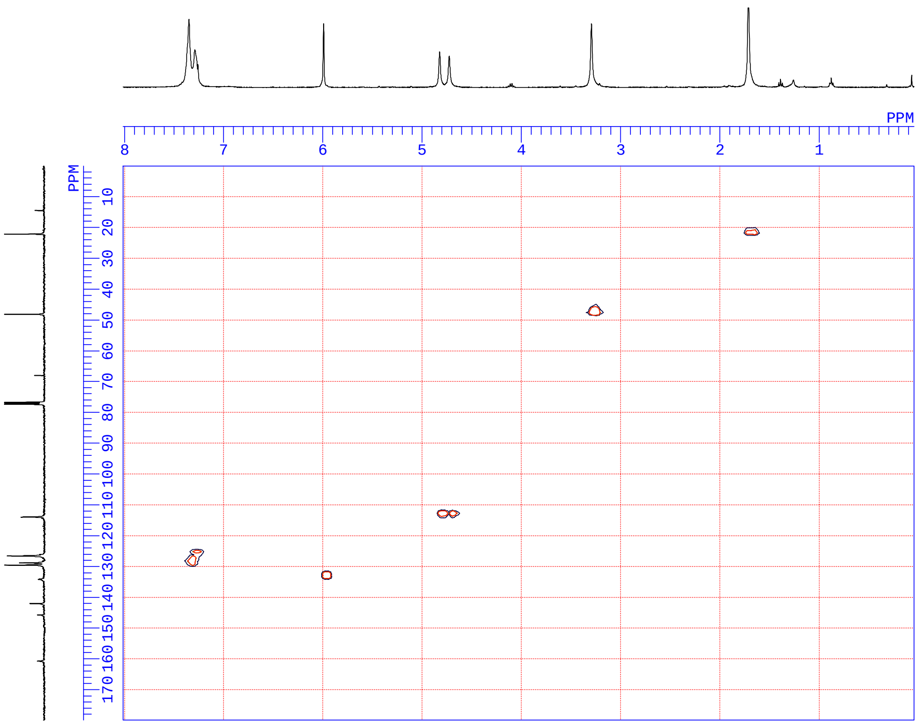
PPM
8
7
6
5
4
3
2
1
PPM
10
20
30
40
50
60
70
80
90
100
110
120
130
140
150
160
170

HMBC

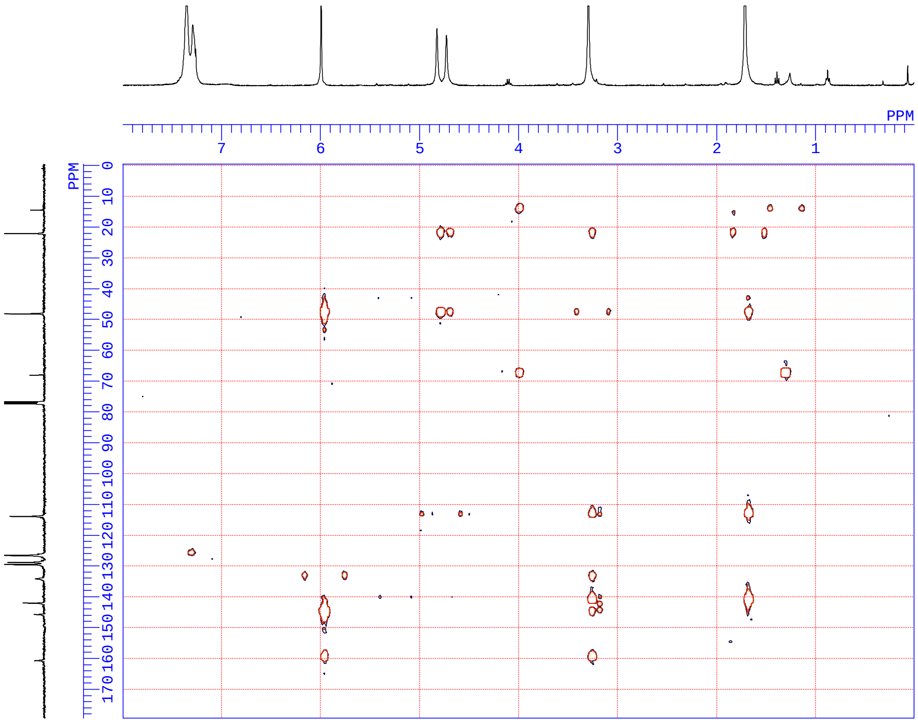
PPM
7
6
5
4
3
2
1
PPM
0
10
20
30
40
50
60
70
80
90
100
110
120
130
140
150
160
170

(Z)-4-methy-1,2-diphenylpenta-1,4-diene (**5**)

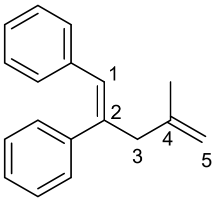

^{1}H NMR (400 MHz, in $CDCl_3$)

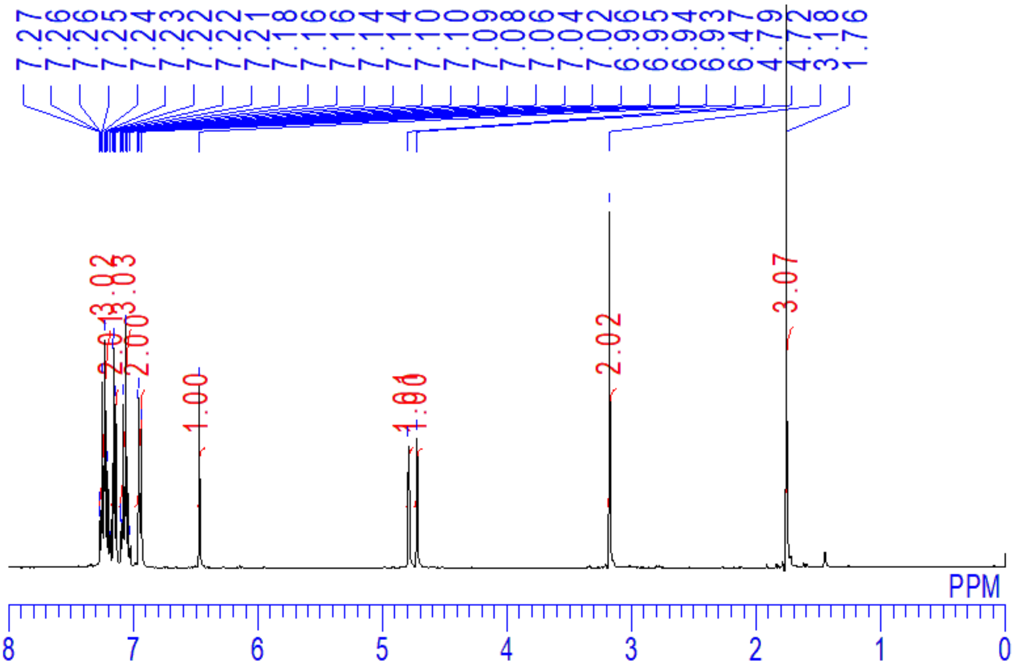

^{13}C NMR (100 MHz, in $CDCl_3$)

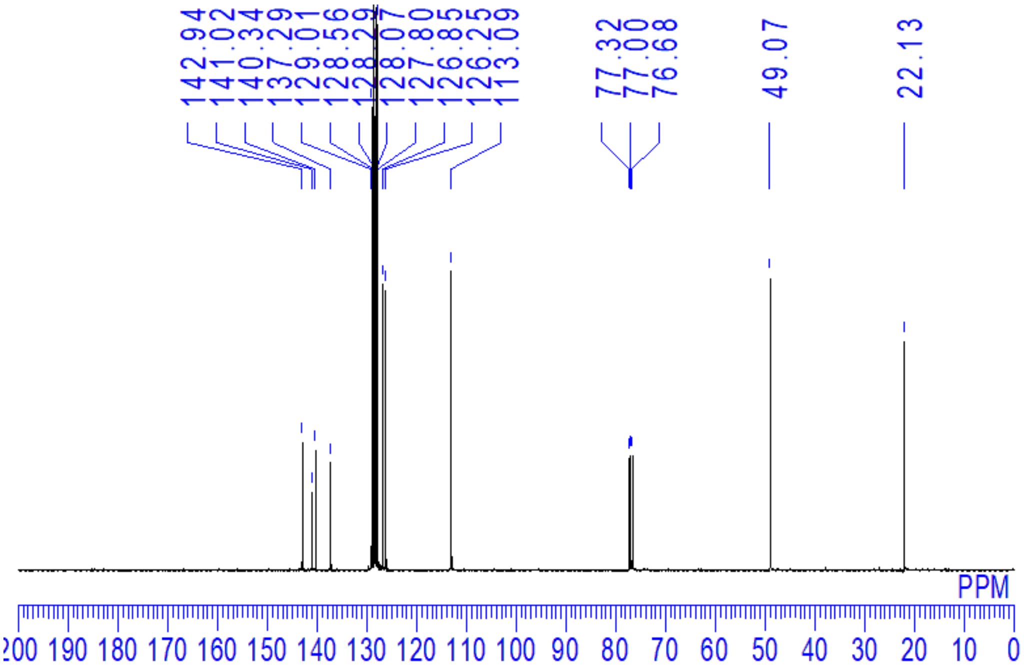

Supplement: Supplementary file 1 [file molecules-23-01884-s001.pdf]
